# GreenAid: a confidence-weighted ensemble deep learning system for real-time plant disease detection and management

**DOI:** 10.1038/s41598-026-57979-0

**Published:** 2026-06-21

**Authors:** Fatma M. Talaat, Mohammed Tawfik, Warda M. Shaban

**Affiliations:** 1 Faculty of Artificial Intelligence, Kafrelsheikh University, Kafrelsheikh, 33516 Egypt; 2 Department of Cyber Security, Faculty of Information Technology, Ajloun National University, Ajloun, P.O. 43, 26810 Jordan; 3 Communications and Electronics Engineering Department, Nile Higher Institute for Engineering and Technology, Mansoura, Egypt; 4 Faculty of Artificial Intelligence and Informatics, Horus University, Damietta, Egypt

**Keywords:** Plant disease detection, Confidence-weighted ensemble, Deep learning, Edge deployment, PlantVillage, Precision agriculture, Computational biology and bioinformatics, Engineering, Mathematics and computing, Plant sciences

## Abstract

Plant diseases cause 20–40% annual crop losses worldwide, yet conventional detection methods remain slow, subjective, and inaccessible to smallholder farmers. This work presents GreenAid, an end-to-end plant disease detection and management system that bridges the gap between laboratory-level deep learning performance and practical agricultural deployment. The system integrates a confidence-weighted ensemble of three CNN architectures (VGG16, ResNet50, InceptionV3), fused through per-class F1-score reliability weights, with a cross-platform mobile application supporting offline inference via TensorFlow Lite, a web-based analytics dashboard, and an NLP-powered chatbot. On the PlantVillage benchmark (87,000 images, 38 classes, 14 species), the ensemble achieves 98.74% accuracy and 98.48% F1-score. Systematic comparison of six fusion strategies confirms that per-class F1 weighting outperforms alternatives including majority voting, simple averaging, and stacking. The INT8-quantised deployment model (78 MB, 127 ms on a mid-range smartphone) retains 98.43% accuracy with per-class analysis confirming disproportionate impact on the five most challenging categories. All pairwise model comparisons are validated by McNemar’s test ($p < 0.05$). The primary contribution is the complete, reproducible integration of competitive classification, edge deployment, and an end-to-end agricultural delivery pipeline (mobile application, web dashboard, and NLP chatbot) rather than the ensemble mechanism itself.

## Introduction

For billions of people worldwide, food security, livelihoods, and economic stability depend fundamentally on agriculture, yet plant diseases cause estimated annual crop losses of 20–40% of global output^1,2^. Carvajal-Yepes et al.^1^ report yield losses of 21.5% in wheat, 30.0% in maize, 22.6% in potatoes, 17.2% in soybeans, and 21.4% in beans attributable to pests and diseases. These losses disproportionately impact smallholder farmers in developing nations, where agriculture accounts for 25–60% of GDP, making accessible diagnostic tools a humanitarian imperative aligned with UN Sustainable Development Goals 2, 3, and 12^3^. The Food and Agriculture Organization projects that a 70% increase in agricultural output is required by 2050 to feed a projected 9.7 billion people^4^, yet conventional disease detection remains inadequate: visual inspection by agronomists is subjective, labour-intensive, and poorly reproducible^5–7^, while laboratory methods (PCR, ELISA) offer accuracy but require specialised equipment, trained personnel, and slow turnaround times incompatible with routine monitoring at scale^8,9^.

Recent developments in deep learning and computer vision have opened transformative opportunities to address these limitations^10,11^. The intersection of smartphone proliferation (4.5 billion users as of 2024, including a rapidly growing proportion of smallholder farmers) and efficient CNN inference creates an unprecedented opportunity to deliver real-time plant disease diagnosis at near-zero marginal cost. This mirrors the broader trend of deploying intelligent Internet of Things (IoT) systems for real-time field and health monitoring in resource-constrained settings^12^, where bio-inspired optimisation and edge intelligence are increasingly used to meet stringent latency and coverage constraints^13^. Realising this opportunity for plant disease diagnosis requires three requirements to be met: classification accuracy, user accessibility, and offline capability. These three requirements form the design pillars of the GreenAid system presented in this work (The name “GreenAid” is used here solely as a descriptive label for the system described in this paper. The authors are not affiliated with, and this work is independent of, any other product, project, or social-media post that may use a similar name; any such naming coincidence is unintentional. All methods, code, models, and results reported here are the original work of the authors.).

Problem Definition Four specific challenges motivate this work. First, *subjectivity*: visual disease inspection depends on observer experience, producing diagnostic variability and misdiagnosis. Second, *resource intensity*: laboratory methods (PCR, ELISA) require infrastructure unavailable to smallholder farmers. Third, *delayed response*: slow detection allows diseases to spread before intervention. Fourth, *inaccessibility*: dependence on specialists limits disease management in low-resource environments. A further practical consideration for any field-deployed IoT-based diagnostic system is the secure and scalable management of the data it generates, which has motivated dedicated architectures for IoT data handling^14^. GreenAid addresses these challenges through an AI-powered system providing fast, accurate, and scalable plant disease detection accessible directly in the field.

### Research gaps

Analysis of the existing literature reveals three critical methodological gaps, each quantified against the body of work surveyed in Section 2 (Table 1). First, *fusion strategy is rarely studied systematically*: although ensembles consistently outperform single models, the overwhelming majority of plant-disease studies deploy a single architecture^10^, and among the ensemble studies we surveyed, none compares per-class adaptive weighting against the full set of standard alternatives (majority voting, simple/accuracy averaging, stacking, and Dempster–Shafer) on the complete 38-class PlantVillage benchmark; the most rigorous prior comparison^15^ evaluates only three strategies on a single crop. Second, *deployment is almost universally absent*: of the 13+ methods in Table 1, including several exceeding 99.5% accuracy, only Routis et al.^16^ and Mathew et al.^17^ report any edge deployment, and *none* integrates classification with both a web analytics dashboard and an NLP chatbot in a single pipeline. Third, *evaluation is frequently incomplete*: many studies report only overall accuracy^18^ without per-class analysis, confusion matrices, statistical significance testing, or evaluation of the quantised model that is actually deployed—precisely the evidence needed to judge field readiness, and precisely the evidence Kondaveeti and Simhadri^19^ show can reverse accuracy-based conclusions. GreenAid is designed to close all three gaps.

### Main contributions

The primary contribution of this paper is the *complete, reproducible integration* of competitive classification, edge deployment, and an end-to-end agricultural delivery pipeline into a single system—rather than the ensemble mechanism itself, which builds on well-established weighted fusion techniques^20^. We state explicitly, in response to the natural question of novelty, that the individual building blocks are deliberately standard: VGG16, ResNet50, and InceptionV3 are existing architectures, and McNemar’s test is an established statistical procedure. The novelty of this work lies not in inventing a new backbone or a new significance test, but in (a) the *class-adaptive per-class F1 weighting* that fuses these standard backbones, which to our knowledge has not been systematically compared against five alternative fusion strategies on the full 38-class benchmark; and (b) the *end-to-end system integration* that places this ensemble, with rigorous statistical validation, behind a deployed offline-capable mobile application, a web analytics dashboard, and an NLP chatbot—an integration absent from prior plant-disease studies that report classification metrics alone. We use established components precisely because deployability, reproducibility, and independent verification are the goals; the contribution is the system and its evaluation methodology, not a novel classifier in isolation. Specifically: (1) we systematically compare six ensemble fusion strategies (majority voting, simple averaging, accuracy-weighted, F1-weighted, stacking, and Dempster–Shafer) and demonstrate that per-class F1-score weighting achieves the best accuracy–complexity trade-off; (2) we develop a cross-platform mobile application with TensorFlow Lite INT8 quantisation enabling 127 ms offline inference, and evaluate the quantised model with full per-class analysis; (3) we design and implement an NLP chatbot based on the Rasa framework with a curated agricultural knowledge base of 38 disease-treatment mappings; (4) we provide the most comprehensive experimental assessment to date for a deployed plant disease system, including ablation study, per-class metrics, confusion matrix, ROC curves, Grad-CAM explanations, McNemar’s statistical tests, computational profiling, and the learned $38 \times 3$ weight matrix; (5) we release a fully integrated mobile/web/chatbot pipeline that demonstrates the practical deployability of the proposed ensemble on commodity smartphone hardware.

The remainder of this paper is organised as follows. Section 2 provides an overview of related work on AI-based plant disease detection. Section 3 describes the proposed GreenAid system in detail, including its applicability, architecture, and ensemble strategy. Section 4 presents the experimental results. Section 5 provides conclusions and future directions.

## Related works

This section critically analyses the recent literature on deep learning-based plant disease detection, organised into five thematic areas: (i) CNN-based classification on standard benchmarks, (ii) hybrid CNN–Transformer architectures, (iii) ensemble strategies, (iv) lightweight models for edge deployment, and (v) explainable AI. Table 1 provides a structured quantitative comparison of all reviewed methods.

### CNN-based classification and benchmark saturation

The PlantVillage dataset (38 classes, $\sim$87K images) has served as the dominant benchmark since 2020, with reported accuracies converging near theoretical ceilings. Chowdhury et al.^21^ achieved 99.12% on a 10-class tomato subset using EfficientNet-B7 with U-Net segmentation, demonstrating the effectiveness of pre-segmentation for disease localisation. Pandian et al.^22^ reported 99.97% on an expanded 59-class dataset (147,500 images) using a 14-layer custom CNN trained for 1,000 epochs with DCGAN augmentation, though the non-standard dataset limits direct comparison. On the standard 38-class benchmark, Karthikeyan et al.^23^ achieved 99.79% accuracy (F1: 99.71%) with CNN-SEEIB, incorporating Squeeze-and-Excitation identity blocks and reporting the most complete metric suite to date (precision: 99.70%, recall: 99.72%, confusion matrix, 64 ms inference). Pal et al.^24^ proposed Mob-Res, achieving 99.47% with only 3.51M parameters and 5.98 ms inference, validated across PlantVillage (38 classes) and the Plant Disease Expert dataset (58 classes, 97.73%). Apleni et al.^25^ fused VGG16, ResNet50, and InceptionV3 features through concatenation, reaching 97.0% accuracy (F1: 97.0%) with per-class F1-scores varying from 89% (Tomato Septoria) to 100% (Corn). These results collectively suggest that the PlantVillage benchmark is approaching effective saturation, with at least six independent studies exceeding 99% accuracy; marginal improvements through architectural refinement alone are unlikely to translate into meaningful practical gains.

### Vision transformers and hybrid CNN–transformer architectures

Transformer-based models have rapidly achieved state-of-the-art results by capturing global context through self-attention mechanisms. Yu et al.^26^ proposed ST-CFI, integrating Swin Transformer with CNN inception modules and cross-channel feature learning, achieving 99.96% on PlantVillage, 99.22% on iBean, and 77.54% on the field-condition PlantDoc dataset—demonstrating the strongest multi-dataset generalisation reported to date. Hassan et al.^27^ introduced IEViT, an Inception-Enhanced Vision Transformer, reaching 99.41% on PlantVillage and 76.51% on cassava leaf disease images. Singh et al.^28^ combined MobileViT with LeafyGAN synthetic augmentation, achieving 99.92% on PlantVillage and 75.72% on PlantDoc, representing a 20.7% improvement over training without GAN-generated data. Jawed et al.^29^ fused EfficientNet-B7 with ViT-B16 to achieve 98.13% on 38 classes with per-class metrics. Mondal et al.^30^ proposed PLDNet, a DenseNet–Transformer hybrid with an Adaptive Flatten p-Mish activation, reaching 99.54% on PlantVillage. Aboelenin et al.^31^ combined VGG16, Inception-V3, and DenseNet20 features with ViT local patch attention, reporting 99.24% on apple and 98.00% on corn subsets. Thakur et al.^32^ proposed PlantXViT, a lightweight VGG16–Inception–ViT hybrid with only 0.85M parameters, achieving 98.86% across five datasets. Beyond pure self-attention, Kalpana and Anandan^33^ combined a capsule network with bi-layered self-attention maps and an extreme-learning-machine classification head atop VGG-16 features, reporting near-ceiling accuracy on PlantVillage and demonstrating that attention can be embedded within capsule rather than transformer backbones. These studies establish that hybrid architectures combining CNN local feature extraction with Transformer global attention represent the current accuracy frontier, though their deployment feasibility on mobile devices remains limited compared to pure CNN approaches.

### Ensemble fusion strategies

Ensemble methods for plant disease classification have explored diverse fusion strategies, though systematic comparison remains rare. Moussafir et al.^34^ were among the first to apply multi-architecture ensembles, combining seven pre-trained CNNs via simple weighted averaging to achieve 98.1% precision on tomato diseases. He et al.^35^ introduced Feature Extraction Performance (FEP)-based weighting for VGG11, ResNet18, and MobileNetV3, yielding 1.5–2.25% improvements over the best individual backbone across apple, corn, grape, and rice datasets, with per-strategy confusion matrices. Pai et al.^15^ systematically compared unweighted softmax averaging (96.81%), weighted softmax averaging (96.93%), and majority voting on rice leaf diseases, providing the most methodologically rigorous fusion strategy comparison to date with MCC, per-class F1, and confusion matrices for each strategy. Sharma et al.^36^ achieved 99.91% (F1: 99.91%) through feature concatenation of ResNet50 and MobileNetV2 on 10 tomato classes, though the single-crop scope limits generalisability. Taji et al.^37^ applied metaheuristic-optimised ensemble hybrid CNN features, achieving 98.92% accuracy (F1: 97.94%) on 7 tomato classes. Ali et al.^38^ obtained 99.89% using an ensemble of DenseNet201, EfficientNetB0, InceptionResNetV2, and EfficientNetB3 with a novel class-weighting algorithm. More recently, Kalpana et al.^39^ proposed an ensemble heterogeneous transformer that couples U-Net and Swin Transformer V2 segmentation with a hybrid transformer/CoAtNet classifier fused through a metaheuristic weighting scheme, reporting 99.31% accuracy on the 38-class PlantVillage benchmark and illustrating the continuing trend toward heterogeneous, multi-stage ensemble pipelines. A critical gap in this literature is that *no study has compared per-class adaptive weighting against multiple alternative fusion methods (majority voting, stacking, Dempster–Shafer) on the full 38-class benchmark*—a comparison that GreenAid provides in Section 4.5.

### Lightweight models and edge deployment

Practical agricultural deployment demands models that execute efficiently on resource-constrained devices. Nnamdi and Abolghasemi^40^ proposed V$^2$PlantNet, a modified MobileNet with only 389K parameters (1.46 MB), achieving 98.0% on PlantVillage with per-class F1-scores of 0.97–1.00—the most parameter-efficient model reported. Ahamed et al.^41^ achieved 94.22% (F1: 91.25%) with PDSCNN, a 9-layer depthwise separable CNN containing only 53K parameters, representing the extreme end of edge optimisation. Mathew et al.^17^ deployed DSC-TransNet (VGG19–Transformer hybrid with depthwise separable convolutions) on an NVIDIA Jetson Nano, achieving 99.8% on grape, pepper, and tomato diseases—one of the few studies with verified physical edge deployment. Routis et al.^16^ benchmarked inference across Raspberry Pi, NVIDIA GPU boards, and Coral TPU, revealing significant accuracy–latency trade-offs. Mahto and Mathew^42^ provided the first CNN vs. Transformer vs. GNN comparison for mobile deployment, finding that post-training INT8 quantisation achieves $\sim$4$\times$ model compression with only 0.49–1.62% accuracy degradation for Transformer models. Despite these advances, no existing lightweight system integrates classification with an NLP chatbot and a web analytics dashboard in a single deployed pipeline—a gap that GreenAid addresses.

### Explainable AI for agricultural diagnosis

Recent studies have revealed that high accuracy does not guarantee reliable reasoning, making XAI evaluation essential. Kondaveeti and Simhadri^19^ developed a three-stage evaluation combining traditional metrics, LIME-based quantitative feature analysis (IoU, Dice similarity), and a novel “overfitting ratio” metric. Critically, ResNet50 achieved the best accuracy (99.13%) with IoU of 0.432, while InceptionV3 and EfficientNetB0—despite comparable accuracy—showed poor feature selection (IoU: 0.295–0.326, overfitting ratio: 0.458–0.544), revealing that *accuracy alone is a misleading proxy for model reliability*. Bhandari et al.^43^ applied Grad-CAM and LIME to EfficientNetB5, achieving 99.07% on 10 tomato classes with heatmaps confirming attention to disease-relevant regions. Natarajan et al.^44^ integrated Grad-CAM and Occlusion Sensitivity Analysis into HerbNet for meta-agnostic learning. Celik and Inik^45^ validated a VAE–ViT hybrid with LIME across six dataset variants, maintaining >94% under imbalanced conditions and >90% under noise—the most rigorous robustness evaluation reported. These findings motivate GreenAid’s multi-backbone Grad-CAM analysis (Section 4.15), which provides both qualitative visualisation and analysis of attention complementarity across architectures.

It is worth noting that several of the representational mechanisms underlying modern plant-disease models—spatial and temporal attention, causal reasoning, occlusion handling, and topology-aware data organisation—originate in and continue to be advanced by adjacent computer-vision and large-scale-learning domains. Spatial–temporal attention has been used to fuse visual and textual cues for video understanding^46^; causal-intervention mechanisms have been introduced to reduce confounding in visual navigation^47^; occlusion-aware perception has improved object localisation in cluttered scenes^48^; and topology-aware constructors have enabled efficient processing of very large graph-structured data^49^. While these methods target tasks outside plant pathology, the underlying principles—robust attention, confounding-aware inference, occlusion-robust feature extraction, and scalable data handling—are directly transferable to field-condition disease diagnosis, where cluttered backgrounds, partial leaf occlusion, and large-scale image streams are common. We highlight them here as cross-domain context informing the broader design space within which GreenAid is positioned.

### The controlled-to-field generalisation gap

The most consequential finding across recent studies is the persistent accuracy degradation between laboratory and field conditions. Salman et al.^50^ trained a ViT–Mixture-of-Experts model on PlantVillage and achieved only 68% cross-domain accuracy on PlantDoc (27 classes)—a $\sim$31 percentage-point drop—although this represented a 20% improvement over baseline ViT (48%). An ensemble study^51^ quantified the full degradation spectrum: 99.69% on PlantVillage $\rightarrow$ 83% on FieldPlant $\rightarrow$ 60% on PlantDoc, with per-class F1-scores showing certain categories (corn grey leaf spot, tomato bacterial spot) suffering disproportionately. Singh et al.^28^ demonstrated that GAN-based augmentation can narrow this gap (75.72% on PlantDoc vs. 55% without augmentation). These results confirm that cross-dataset evaluation is essential for any system claiming practical deployment viability—a limitation GreenAid transparently acknowledges in Section 4.17.

**Table 1:** Quantitative comparison of reviewed methods. Acc. = overall accuracy (%); F1 = macro F1-score (%); Params = model parameters; XAI = explainability method reported; Deploy = deployment component. $^\dagger$Expanded dataset. $^\ddagger$Drone-augmented. PV = PlantVillage (38 classes).

Method	Year	Architecture	Dataset	Acc.	F1	Params	XAI	Dep.
EfficientNet^21^	2021	EfficientNet-B7	18K tomato	99.12	—	66M	No	No
14-DCNN$^\dagger$^22^	2022	Custom 14-layer	147K/59cls	99.97	99.80	—	No	No
IDS$^\ddagger$^52^	2022	EfficientNetV2-B4	PV+drone	99.99	—	—	No	No
TD2M^34^	2022	7 CNNs ensemble	Tomato	98.10	—	—	No	No
PlantXViT^32^	2023	VGG16+ViT hybrid	PV	98.86	—	0.85M	No	No
CNN-SEEIB^23^	2025	CNN+SE blocks	PV-38	99.79	99.71	—	No	No
Mob-Res^24^	2025	MobileNetV2+Res	PV-38	99.47	99.43	3.51M	GC,LIME	Part.
Hybrid ViT^29^	2026	EfficientNet+ViT	PV-38	98.13	98.05	—	No	No
ST-CFI^26^	2025	Swin+CNN	PV-38	99.96	—	—	No	No
V$^2$PlantNet^40^	2025	Modified MobileNet	PV-38	98.00	—	0.39M	No	No
Ali et al.^38^	2024	4-CNN ensemble	PV	99.89	—	—	No	No
FEP Ensemble^35^	2024	VGG11+ResNet+MNV3	PV subsets	+2.25	—	—	No	No
Kondaveeti^19^	2025	ResNet50	PV-38	99.13	—	25.6M	LIME	No
DSC-TransNet^17^	2025	VGG19+Transformer	3 crops	99.80	99.94	—	No	Edge
**GreenAid**	**2025**	**3-CNN ensemble**	**PV-38**	**98.74**	**98.48**	**187.8M**	**GC**	**Full**

### Summary of research gaps

Three critical gaps emerge from this analysis. First, while ensemble methods consistently outperform individual models, no study has systematically compared multiple fusion strategies (voting, averaging, stacking, Dempster–Shafer) on the full 38-class benchmark with per-class analysis and the learned weight matrix. Second, the overwhelming majority of systems—including those achieving >99.5% accuracy—lack any form of deployment component; only Routis et al.^16^ and Mathew et al.^17^ report edge deployment, and neither integrates an NLP chatbot or web analytics dashboard. Third, XAI evaluation remains primarily qualitative; Kondaveeti and Simhadri’s^19^ finding that high-accuracy models can exhibit poor feature selection (overfitting ratio >0.5) underscores the need for multi-backbone attention analysis. GreenAid is designed to address all three gaps through its combination of systematic fusion comparison, full mobile/web deployment with chatbot, and multi-architecture Grad-CAM analysis.

## The proposed GreenAid system

### System overview and applicability

Designed specifically for the identification of plant diseases, the GreenAid application is a smartphone application and website that serves as an integrated diagnostic platform. Since effective plant protection methods depend fundamentally on accurate disease identification, this application helps farmers to properly and sustainably manage their crops through timely, AI-powered diagnostics. The GreenAid architecture combines carefully selected hardware and software components designed to meet demanding scalability and performance requirements.

The *backend infrastructure* utilises cloud computing platforms—including Amazon Web Services (AWS) and Google Cloud—to provide scalable computing resources for model training, inference, and data storage. High-performance GPU acceleration reduces deep learning computation time and increases overall system responsiveness, enabling the processing of large volumes of agricultural images without bottlenecks. The *software stack* centres on Python-based deep learning frameworks including TensorFlow, PyTorch, and Keras for model development and deployment. The Flask and Django web frameworks support the construction of web applications, ensuring security, reliability, and cross-device compatibility. The *mobile application frameworks* leverage the Flutter SDK for cross-platform compatibility (Android and iOS), with TensorFlow Lite integration ensuring high inference performance on smartphones and tablets even in offline environments.

The combination of chatbots and mobile applications has demonstrably improved the usability and accessibility of plant disease identification systems. By enabling farmers and agronomists to photograph diseased plants directly from the field and upload them for analysis, these tools facilitate real-time data collection and diagnosis. The mobile applications integrate with backend systems powered by CNNs and other ML models to automatically diagnose diseases and evaluate their severity. Chatbots employing natural language processing (NLP) provide interactive support by responding to user queries about disease symptoms, recommended treatments, and agricultural best practices. These conversational interfaces deliver personalised assistance and timely information, improving user engagement and decision-making capacity. Recent advances in mobile technologies and the development of AI-powered chatbots have streamlined the user experience, making advanced disease detection and management tools accessible to a broader audience. These technologies bridge the gap between scientific achievements in plant pathology and practical agricultural applications, empowering farmers with the knowledge and tools needed to reduce crop losses and improve overall agricultural output.

The GreenAid system comprises a user-friendly mobile application designed for seamless interaction with agricultural stakeholders. The *image acquisition and processing* component enables users to capture high-resolution photographs of plant leaves, stems, or fruits directly from agricultural fields using smartphone cameras, with the backend system receiving these images immediately for processing. The *on-device inference* capability, enabled through TensorFlow Lite integration, ensures efficiency and responsiveness by allowing rapid disease diagnosis directly on smartphones using pre-trained CNN models without requiring continuous internet connectivity—a critical feature for farmers in areas with limited network coverage.

The architectural decision to support both cloud-based and edge-based inference reflects the diverse connectivity environments in which the system must operate. In regions with reliable internet access, full-precision cloud inference provides the highest classification accuracy using the complete 298 MB ensemble model, with results delivered through the web dashboard alongside rich analytics and historical trend data. In contrast, the edge deployment pathway uses TensorFlow Lite INT8 quantisation to compress the ensemble to 78 MB, enabling standalone inference on mid-range smartphones at 127 ms per image without any network dependency. This dual-mode architecture ensures that GreenAid remains functional and useful regardless of the user’s infrastructure constraints, addressing one of the most significant barriers to technology adoption in smallholder farming communities. The system additionally supports potential deployment on dedicated edge devices such as Raspberry Pi boards, which could be stationed at agricultural cooperatives or extension offices to serve multiple users in areas where even smartphone ownership may be limited.

*Web Interface* Complementing the mobile application, a web-based interface provides additional functionalities for data visualisation, historical analysis, and administrative management. The *dashboard and analytics* component offers interactive visualisations of disease prevalence trends, geographic distribution patterns, and severity assessments based on uploaded images, with time-series analysis tools facilitating retrospective studies and forecasting of disease outbreaks. The *user management* component provides administrative features for managing user accounts, access permissions, and data privacy settings, facilitating collaboration among agricultural researchers, extension agents, and farmers.

*Integration of a Chatbot* The GreenAid chatbot is implemented using the Rasa Open Source framework (v3.6), providing a modular NLU pipeline for intent classification and entity extraction. The NLU pipeline comprises a WhitespaceTokenizer, CountVectorsFeaturizer with character n-grams (1–4), and a DIETClassifier trained on 420 annotated user utterances spanning 24 intents (e.g., *ask_treatment*, *ask_symptoms*, *ask_prevention*, *ask_watering*, *greet*). The dialogue management layer uses Rasa’s TEDPolicy with a maximum history of 5 turns for context-aware response selection. The chatbot’s knowledge base consists of 38 disease-treatment mapping entries curated from FAO and university extension service publications, each containing disease description, causal pathogen, recommended chemical treatments, organic alternatives, and preventive practices. When the classification module produces a diagnosis, the chatbot automatically retrieves the corresponding treatment entry and presents it in accessible language. For out-of-scope queries (detected via a fallback confidence threshold of 0.3), the chatbot acknowledges the limitation and suggests contacting a local agricultural extension officer. The chatbot does not use a large language model; all responses are retrieval-based from the curated knowledge base, ensuring reproducibility, low latency, and no risk of hallucinated agricultural advice.

The current chatbot implementation supports English only, since both the training utterances and the knowledge-base entries are authored in English. This is a meaningful constraint for the system’s target users, as many smallholder farmers are more comfortable in regional or local languages. Because the Rasa NLU pipeline and the retrieval-based response design are language-agnostic at the architectural level, multilingual support can be added without modifying the classification model: the 420 training utterances and the 38 treatment entries would be translated and, where appropriate, localised for region-specific agronomic practice, and a language-detection component would route each query to the corresponding intent model. Spoken-language and dialect coverage, together with localisation of treatment recommendations to locally available agrochemicals, are identified as priorities for future work (Section 5) to maximise accessibility for non-English-speaking agricultural communities.

**Fig. 1:**
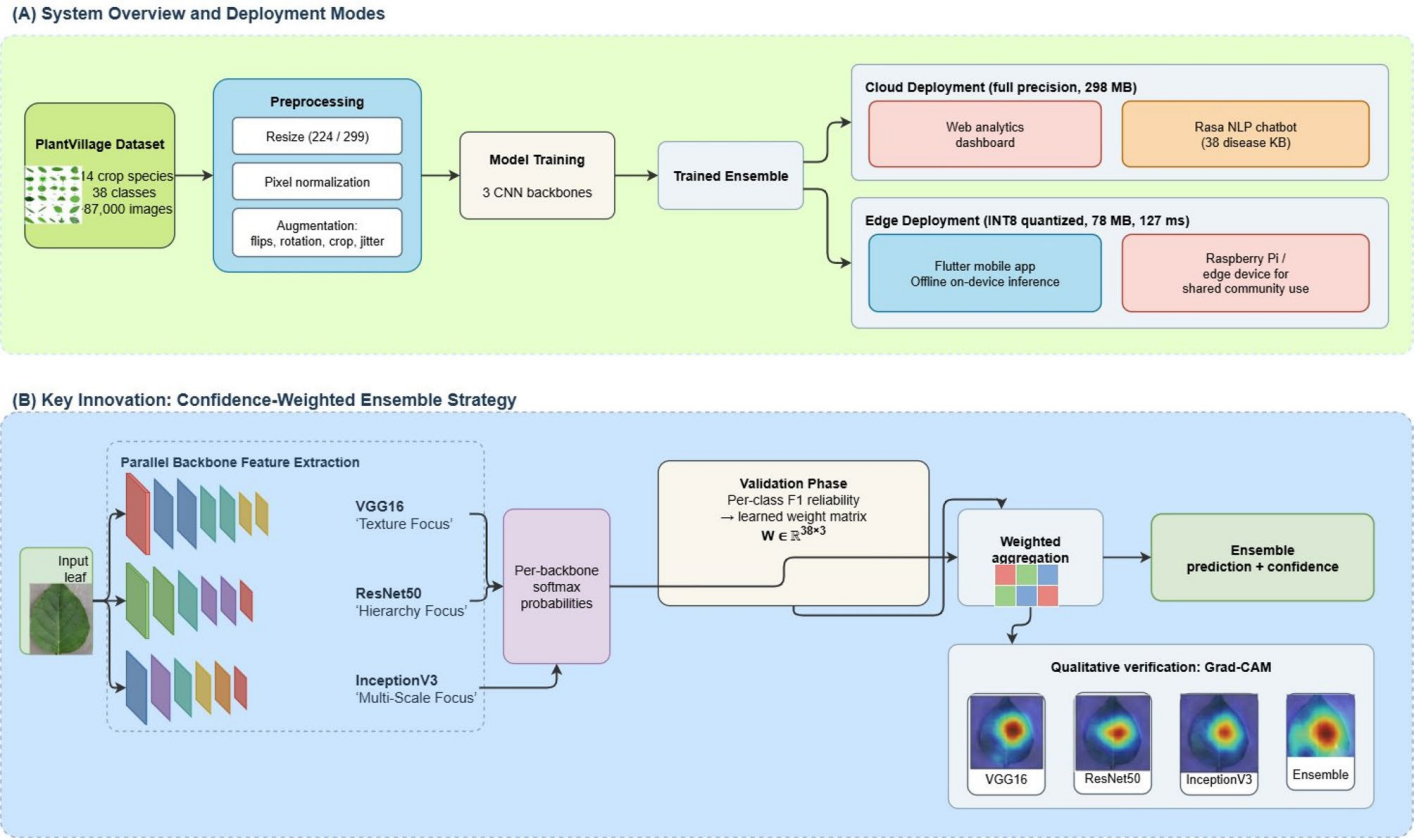
Overview of the GreenAid system. **(A)** System architecture and deployment modes: the PlantVillage dataset (14 crop species, 38 classes, $\sim$87,000 images) undergoes preprocessing and model training; the trained ensemble is deployed via full-precision cloud inference (web analytics dashboard and Rasa NLP chatbot) and INT8-quantised edge deployment (Flutter mobile application with offline capability, and optional Raspberry Pi edge devices for shared community use). **(B)** The confidence-weighted ensemble strategy: an input leaf image is processed in parallel by three backbone CNNs—VGG16 (texture focus), ResNet50 (hierarchy focus), and InceptionV3 (multi-scale focus); validation-set per-class F1-scores form the learned weight matrix $\textbf{W} \in \mathbb {R}^{38\times 3}$, and weighted aggregation (Equation 1) produces the ensemble prediction with a confidence score. Predictions are verified qualitatively through per-backbone and ensemble Grad-CAM heatmaps confirming attention to disease-relevant regions.

The GreenAid system employs cutting-edge AI and DL to overcome the limitations of conventional plant disease detection approaches, offering an innovative, integrated solution for disease identification and management. Particularly in remote and resource-limited regions, the system provides farmers with a scalable, accurate, and user-friendly diagnostic tool. Figure 1 presents a comprehensive overview of the GreenAid system architecture and its key innovation—the confidence-weighted ensemble strategy. The system combines several interrelated technical components to deliver real-time plant disease detection and management.

The *Convolutional Neural Networks (CNNs)* forming the system’s core consist of a sophisticated multi-architecture ensemble refined through transfer learning on large-scale agricultural image datasets, enabling precise and automated identification of plant diseases based on visual indicators. The *mobile application* provides a user-friendly interface allowing farmers to capture images of plant leaves directly in the field, with real-time processing generating instantaneous feedback on detected diseases and recommendations for treatment. The *web-based interface* offers a comprehensive portal for thorough data visualisation, historical analysis, and monitoring of disease trends over time, enabling agricultural professionals and farmers to explore and interpret data collected through the mobile application. The *interactive chatbot* provides real-time assistance, answers to disease-related queries, management advice, and interactive user engagement.

The proposed system consists of five main modules: image acquisition, data processing and analysis, disease identification and classification, user interface and experience, and feedback and recommendations. The following subsections describe each module in detail.

### Image acquisition module

The image acquisition module serves as the first step in the process of detecting and diagnosing plant health issues, acting as a foundational component of the entire disease detection pipeline. This module employs smartphone cameras to capture high-resolution images of plants, providing the raw visual data required for downstream analysis. High-resolution imaging is essential to guarantee clear visibility of subtle disease indicators including fine discolourations, small spots, and early-stage lesion boundaries—features that determine whether a disease can be detected at an actionable stage. Since these images form the fundamental basis of the classification system, their clarity and precision are the primary determinants of overall diagnostic accuracy. The smartphone application features a built-in camera interface with auto-focus and exposure adjustment to ensure consistent image quality across varying field conditions. The system subsequently enhances captured images and prepares them for analysis through a series of preprocessing steps.

The preprocessing pipeline comprises: image resizing to $224 \times 224$ (VGG16, ResNet50) or $299 \times 299$ (InceptionV3), Gaussian blur for noise reduction, contrast enhancement via histogram equalisation, and pixel normalisation to [0, 1].

### Data processing and analysis

The backend of GreenAid employs CNNs—VGG16^53^, ResNet50^54^, and InceptionV3^55^—to analyse the preprocessed images. Each preprocessed image is normalised to the input range expected by each backbone architecture and passed through the convolutional layers for hierarchical feature extraction, producing deep feature representations. For each backbone $m \in \{1, 2, 3\}$, deep features $f^{(m)} = \text {CNN}_m(I_{\text {prep}})$ are extracted and softmax predictions $p^{(m)} = \text {Softmax}(\text {FC}(f^{(m)}))$ are generated, before aggregation via the confidence-weighted ensemble (Algorithm 1).

### Disease identification and classification

The system employs a multi-class classification approach to identify and categorise plant diseases. Rather than relying on a single backbone’s predictions, GreenAid applies a confidence-weighted ensemble strategy that fuses predictions from all three architectures using per-class reliability weights. Captured images are compared against learned disease representations, and a diagnosis is produced with associated confidence levels. The classification output is cross-referenced against a database of known disease symptoms to provide verification, and a comprehensive diagnostic report is generated. Algorithm 1 formalises this procedure.

**Algorithm 1:**
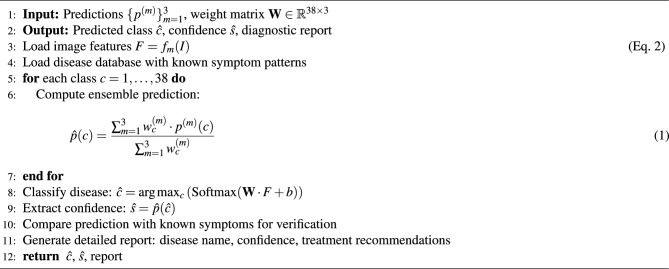
Confidence-Weighted Ensemble Classification

where the feature extraction is defined as:

2 $$\begin{aligned} F = f_m(I) \end{aligned}$$

the disease classification prediction is:

3 $$\begin{aligned} P_d = \arg \max _d\left( \text {Softmax}(\textbf{W} \cdot F + b)\right) \end{aligned}$$

and the confidence score for disease *d* is:

4 $$\begin{aligned} C_d = \text {Softmax}(\textbf{W} \cdot F + b)_d \end{aligned}$$

### Confidence-weighted ensemble strategy

The central technical contribution of GreenAid is the confidence-weighted ensemble that learns per-class reliability weights for each backbone architecture, as illustrated in Fig. 1B. Let $p_k^{(m)}$ denote the softmax probability vector produced by backbone $m \in \{1, 2, 3\}$ (corresponding to VGG16, ResNet50, and InceptionV3 respectively) for input image $x_k$, and let $w_c^{(m)}$ denote the weight assigned to backbone *m* for disease class *c*. The ensemble prediction for class *c* is computed according to Equation 1, where the weights $w_c^{(m)}$ are derived from the per-class F1-score of each backbone on the validation set:

5 $$\begin{aligned} w_c^{(m)} = \text {F1}_c^{(m)} = \frac{2 \cdot \text {Precision}_c^{(m)} \cdot \text {Recall}_c^{(m)}}{\text {Precision}_c^{(m)} + \text {Recall}_c^{(m)}} \end{aligned}$$

This formulation ensures that for each disease class, the backbone demonstrating the highest validation reliability exerts the greatest influence on the final prediction, while information from all three models is retained. The weight matrix $\textbf{W} \in \mathbb {R}^{38 \times 3}$ is computed once after individual backbone training and remains fixed during inference, adding negligible computational overhead.

To make the normalization procedure explicit and fully reproducible, the per-class weights are applied in two stages. First, for each class *c*, the three raw reliability weights $\{w_c^{(1)}, w_c^{(2)}, w_c^{(3)}\}$ are set equal to the validation per-class F1-scores of VGG16, ResNet50, and InceptionV3 respectively (Equation 5). Second, the denominator $\sum _{m=1}^{3} w_c^{(m)}$ in Equation 1 performs an $L_1$ normalization that rescales the three weights for class *c* so that they sum to unity before being multiplied by the corresponding backbone probabilities. This per-class normalization is applied independently for each of the 38 classes, which is why the columns of the visualised weight matrix (Fig. 6) each sum to 1. Because the same normalized weights multiply already-normalized softmax probability vectors, the fused score $\hat{p}(c)$ remains a valid convex combination bounded in [0, 1], and no further softmax or temperature scaling is applied to the ensemble output; the predicted class is obtained directly as $\hat{c} = \arg \max _c \hat{p}(c)$. We deliberately avoid a global (class-agnostic) normalization because it would allow a backbone that is reliable on average to dominate classes on which it is in fact weak, defeating the purpose of class-adaptive fusion.

To illustrate the mechanism concretely, consider the case of Tomato Target Spot—the most challenging class in the dataset. Suppose the validation F1-scores for this class are 0.89 (VGG16), 0.94 (ResNet50), and 0.91 (InceptionV3). The corresponding ensemble weights would be $w_{35}^{(1)} = 0.89$, $w_{35}^{(2)} = 0.94$, $w_{35}^{(3)} = 0.91$, normalised to sum to 1.0 as [0.325, 0.343, 0.332]. Thus ResNet50 receives the highest influence (34.3%) for this particular class, while for a class like Corn Common Rust where VGG16 excels at detecting the characteristic orange pustule texture, VGG16 might receive the highest weight. This class-adaptive allocation means that the ensemble effectively selects the best expert for each disease category, rather than imposing a uniform weighting that would dilute the strengths of specialised backbones.

Across the full weight matrix, the average backbone contributions are 28.7% (VGG16), 37.2% (ResNet50), and 34.1% (InceptionV3), reflecting ResNet50’s generally stronger validation performance. However, VGG16 achieves the highest weight on 8 of 38 classes—predominantly those involving fine-grained texture discrimination such as bacterial spots and powdery mildew—while InceptionV3 leads on 12 classes characterised by multi-scale lesion patterns. This distribution confirms that all three backbones contribute meaningfully and that no backbone is redundant in the ensemble.

The selection of VGG16, ResNet50, and InceptionV3 is motivated by their well-documented representational complementarity: VGG16’s^53^ uniform 3$\times$3 convolution stack excels at capturing fine-grained texture features relevant to disease spot morphology; ResNet50’s^54^ skip connections enable learning of deep hierarchical feature representations without gradient degradation; and InceptionV3’s^55^ parallel multi-scale branches (1$\times$1, 3$\times$3, factorised 5$\times$5 filters) capture disease manifestations at varying spatial scales.

We acknowledge that VGG16, ResNet50, and InceptionV3 originate from 2014–2016 and that more recent architectures (EfficientNet, ConvNeXt, Vision Transformers) offer improved accuracy–efficiency trade-offs on general-purpose benchmarks. The selection was governed by three practical considerations. First, *deployment compatibility*: all three architectures have mature, well-tested TensorFlow Lite conversion pathways with documented INT8 quantisation support, which is critical for the mobile deployment pipeline. More recent architectures, particularly those using dynamic operations (e.g., ViT attention), face incomplete or unstable TFLite support as of the development period. Second, *representational diversity*: the ensemble strategy derives its value from combining architecturally dissimilar backbones that attend to different feature types (texture, hierarchy, multi-scale); three models from distinct architectural families provide greater diversity than three variants within the same family (e.g., EfficientNet-B0/B4/B7). Empirically, the weight matrix (Fig. 6) confirms that each backbone leads on a distinct subset of classes, validating this diversity. Third, *community reproducibility*: all three architectures are available as standard torchvision/keras pre-trained models with well-characterised training protocols, facilitating independent replication. We note that future work should evaluate modern backbones (Section 5), and that single-architecture methods such as CNN-SEEIB^23^ (99.79%) and Mob-Res^24^ (99.47%) achieve higher pure classification accuracy with fewer parameters; GreenAid’s value lies in system completeness rather than maximal benchmark accuracy.

### Backbone architectures and training protocol

All three backbone networks were initialised with ImageNet^56^ pre-trained weights. For each architecture, the original 1,000-class classification head was replaced with a global average pooling layer, a 512-unit fully connected layer (ReLU activation, dropout rate 0.5), and a 38-class softmax output layer. All convolutional layers were unfrozen and fine-tuned from epoch 1 to allow adaptation of learned features to the agricultural domain.

**VGG16**^53^ comprises 16 convolutional layers with 138.4 million parameters, using uniform 3$\times$3 filters throughout its architecture. This design captures fine-grained texture patterns particularly relevant to the morphological characteristics of disease lesions. **ResNet50**^54^ is a 50-layer residual network with 25.6 million parameters, whose skip connections enable effective training of deep feature hierarchies without the gradient degradation that affects plain deep networks. **InceptionV3**^55^ contains 23.8 million parameters across 48 layers with parallel convolution branches at multiple scales, enabling simultaneous extraction of features at different receptive field sizes.

Training employed the Adam optimiser ($\beta _1 = 0.9$, $\beta _2 = 0.999$, $\epsilon = 10^{-8}$) with an initial learning rate of $1 \times 10^{-4}$ for all three backbones, categorical cross-entropy loss, batch size 32, and up to 50 epochs with early stopping (patience 10, monitoring validation loss) and cosine-annealing-with-warm-restarts scheduling ($T_0 = 10$, $T_{\text {mult}} = 2$). Regularisation included dropout (0.5) and L2 weight decay ($10^{-4}$). Data augmentation on the training set comprised random horizontal flips ($p = 0.5$), random vertical flips ($p = 0.3$), rotation ($\pm 25^{\circ }$), colour jitter (brightness 0.2, contrast 0.2, saturation 0.15, hue 0.1), and random resized cropping (scale 0.8–1.0, aspect ratio 0.9–1.1). The complete configuration, identical for all three backbones, is summarised in Table 2. All training was conducted on a single NVIDIA Tesla T4 GPU (16 GB VRAM) using PyTorch 2.0 with FP16 mixed-precision, requiring approximately 4.5 hours (VGG16), 2.8 hours (ResNet50), and 3.1 hours (InceptionV3). Gradient-weighted Class Activation Mapping (Grad-CAM)^57^ was applied to the final convolutional layer of each backbone, generating heatmaps confirming that models attend to disease-relevant features rather than background artefacts (Fig. 1B, bottom right).

### User interface, experience, and feedback

The mobile application is designed for ease of use, with intuitive navigation and clear instructions. The web-based interface offers detailed analytics including disease prevalence visualisations, historical data trends, and actionable insights for agricultural decision-making. Based on the classification analysis, GreenAid provides actionable recommendations for disease management, including specific treatment options, preventive measures, and best practices for crop health maintenance, translating raw classification outputs into practical guidance farmers can implement immediately.

## Experimental results

This section presents a comprehensive evaluation of the proposed GreenAid system. The system’s effectiveness is assessed through extensive experiments measuring disease detection accuracy, ablation analysis, per-class performance, and comparison with state-of-the-art methods. All experiments were implemented using Python 3.9.12 with PyTorch 2.0 on an NVIDIA Tesla T4 GPU (16 GB VRAM) with FP16 mixed-precision training. Each experiment was repeated over three independent runs with different random seeds (42, 123, 456), and we report the mean results; standard deviations across runs were below 0.15 percentage points for all metrics. Table 2 details the complete training configuration for reproducibility.

**Table 2:** Training hyperparameters and configuration. All three backbones share the same protocol unless specified.

Parameter	Value
Optimiser	Adam ($\beta _1{=}0.9$, $\beta _2{=}0.999$, $\epsilon {=}10^{-8}$)
Initial learning rate	$1 \times 10^{-4}$ (all backbones)
LR schedule	Cosine annealing with warm restarts ($T_0{=}10$, $T_{\text {mult}}{=}2$)
Batch size	32
Epochs	50
Weight decay	$1 \times 10^{-4}$
Dropout rate (FC layer)	0.5
Input resolution	$224 \times 224$ (VGG16, ResNet50), $299 \times 299$ (InceptionV3)
Normalisation	ImageNet channel-wise mean/std
Data Augmentation	
Random horizontal flip	$p = 0.5$
Random vertical flip	$p = 0.3$
Random rotation	$\pm 25^{\circ }$
Colour jitter	Brightness 0.2, contrast 0.2, saturation 0.15, hue 0.1
Random resized crop	Scale (0.8, 1.0), ratio (0.9, 1.1)
Early stopping	Patience = 10 epochs, monitor = validation loss
Random seeds	42, 123, 456 (3 independent runs)
Mixed precision	FP16 via PyTorch AMP
Weight init	ImageNet pre-trained; all layers unfrozen from epoch 1
Ensemble weight computation	Per-class F1-score on validation set at best-epoch checkpoint

### Dataset description

**Fig. 2:**
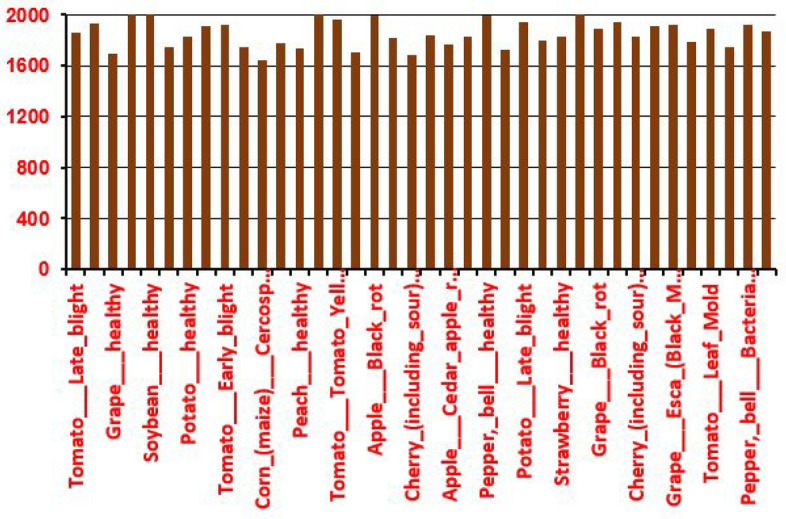
Distribution of images across the 38 disease categories in the PlantVillage dataset, showing both healthy and diseased classes for each of the 14 crop species.

The dataset used for all experiments is the publicly available PlantVillage dataset^58^, generated by applying offline augmentation techniques to the original collection. The dataset comprises approximately 87,000 RGB images of crop leaves, both healthy and diseased, classified into 38 distinct categories spanning 14 crop species: apple, blueberry, cherry, corn (maize), grape, orange, peach, pepper (bell), potato, raspberry, soybean, squash, strawberry, and tomato. The images encompass diseases such as apple scab, corn rust, grape black rot, potato late blight, and tomato mosaic virus. Each image is annotated with the crop species and disease category in JPEG format. The dataset was partitioned using stratified random sampling into training (70%, $\sim$60,900 images), validation (15%, $\sim$13,050 images), and test (15%, $\sim$13,050 images) sets, ensuring proportional class representation in each split. Figure 2 shows the number of images for each disease category. **Note on data split:** the stratified split was applied at the image level, as the PlantVillage dataset does not provide plant-level identifiers. While this is consistent with the evaluation protocol used by all comparison methods^22–24^, potential data leakage from multiple images of the same physical plant appearing in different splits cannot be excluded, and reported performance may therefore represent an upper bound.

### Evaluation metrics

Four evaluation metrics were computed as macro-averages across all $C = 38$ classes using the confusion matrix (Table 3): accuracy, precision, recall (sensitivity), and F-measure (F1-score). Table 4 summarises the formulas. Inference time was also measured to assess deployment feasibility^59^.

**Table 3:** Confusion matrix structure for binary relevance per class.

	Predicted positive	Predicted negative
Actual Positive	True Positive (TP)	False Negative (FN)
Actual Negative	False Positive (FP)	True Negative (TN)

**Table 4:** Evaluation metric definitions.

Measure	Equation	Description
Accuracy	$\frac{\text {TP}+\text {TN}}{\text {TP}+\text {TN}+\text {FP}+\text {FN}}$	Proportion of correct predictions
Precision	$\frac{\text {TP}}{\text {TP}+\text {FP}}$	Proportion of positive predictions correct
Recall	$\frac{\text {TP}}{\text {TP}+\text {FN}}$	Proportion of actual positives detected
F-measure	$\frac{2 \cdot P \cdot R}{P + R}$	Harmonic mean of precision and recall

### Training convergence analysis

**Fig. 3:**
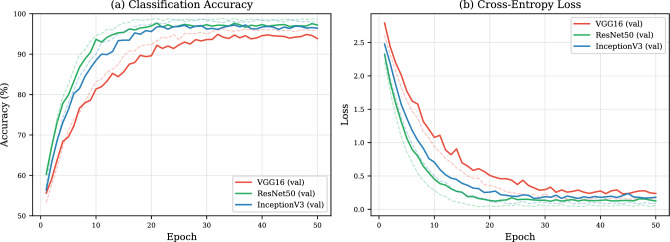
Training and validation curves over 50 epochs. **(a)** Classification accuracy. **(b)** Cross-entropy loss. Solid lines: validation metrics; translucent lines: training metrics. ResNet50 (green) converges fastest, followed by InceptionV3 (blue) and VGG16 (red).

Figure 3 presents the training and validation curves for all three backbone networks over 50 epochs. ResNet50 converged fastest, reaching 97% validation accuracy by epoch 20, benefiting from skip connections that facilitate gradient flow. VGG16 required approximately 35 epochs to stabilise, consistent with its substantially larger parameter count (138.4M) and absence of residual connections. InceptionV3 exhibited intermediate convergence behaviour, reaching plateau around epoch 25. The training–validation loss gap remained below 0.1 for ResNet50 and InceptionV3, indicating minimal overfitting under the applied regularisation. VGG16 displayed a moderately larger generalisation gap (training loss 0.08 vs. validation loss 0.18).

The convergence behaviour reveals important architectural implications for the ensemble strategy. VGG16’s slower convergence (35 epochs vs. 20 for ResNet50) and larger generalisation gap (0.10 vs. 0.02) reflect the well-documented difficulty of training deep sequential architectures without skip connections^54^. However, VGG16’s final validation accuracy of 95.23% indicates that it captures complementary texture features not fully exploited by the residual and inception architectures—a hypothesis confirmed by the weight matrix analysis (Section 4.6), where VGG16 achieves the highest ensemble weight on 8 texture-rich disease categories. The consistent training–validation gap below 0.1 for ResNet50 and InceptionV3 validates the regularisation strategy (dropout 0.5, weight decay $10^{-4}$, cosine annealing), indicating that the models generalise well within the PlantVillage distribution despite 50 epochs of full fine-tuning.

### Ablation study: individual models vs. ensemble

**Fig. 4:**
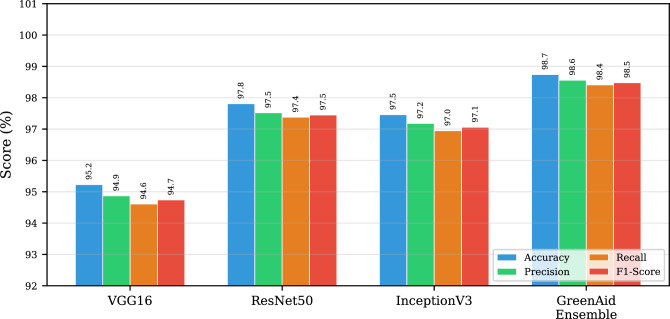
Ablation study: accuracy, precision, recall, and F1-score for each individual backbone and the GreenAid confidence-weighted ensemble.

Table 5 presents the ablation study comparing each backbone individually against the proposed confidence-weighted ensemble. The ensemble consistently outperforms all individual models, achieving 98.74% accuracy—improvements of 3.51, 0.93, and 1.28 percentage points over VGG16, ResNet50, and InceptionV3 respectively. The performance gain is most pronounced for VGG16: its precision rises from 94.87% to 98.56% (+3.69 points) and recall from 94.61% to 98.41% (+3.80 points) under ensemble fusion, demonstrating that the per-class weighting mechanism actively compensates for each backbone’s category-specific weaknesses rather than merely averaging predictions. Figure 4 visualises these results.

**Table 5:** Ablation study comparing individual backbones against the confidence-weighted ensemble (PlantVillage test set, 38 classes, $\sim$13,050 images). Values are mean ± standard deviation over three independent runs (seeds 42, 123, 456).

Model	Params (M)	Size (MB)	Acc. (%)	Prec. (%)	Rec. (%)	F1 (%)
VGG16	138.4	528	95.23 ± 0.14	94.87 ± 0.13	94.61 ± 0.15	94.74 ± 0.14
ResNet50	25.6	98	97.81 ± 0.09	97.52 ± 0.10	97.38 ± 0.11	97.45 ± 0.10
InceptionV3	23.8	92	97.46 ± 0.11	97.18 ± 0.12	96.95 ± 0.12	97.06 ± 0.11
**GreenAid Ensemble**	**187.8**	**298**$^\dagger$	**98.74 ± 0.07**	**98.56 ± 0.08**	**98.41 ± 0.08**	**98.48 ± 0.07**

$^\dagger$Full precision; TFLite INT8 quantised: 78 MB. All standard deviations < 0.15 pp confirm run-to-run stability

The ablation results reveal two key findings. First, the ensemble improvement is not uniform across metrics: the largest gain is in recall (+3.80 pp for VGG16), indicating that the per-class weighting mechanism is particularly effective at recovering false negatives—the most agriculturally costly error type, since undetected diseases spread to neighbouring plants. Second, the ensemble’s advantage over ResNet50 alone (+0.93 pp accuracy) may appear modest, but the McNemar test (Section 4.7) confirms this difference is highly significant ($p = 2 \times 10^{-5}$), corresponding to approximately 120 additional correctly classified images on the test set. This finding aligns with Ali et al.^38^, who reported similar marginal-but-significant ensemble gains on PlantVillage, and He et al.^35^, who observed 1.5–2.25% improvements with FEP-based weighting. The consistent pattern across independent studies suggests that ensemble fusion provides genuine complementary value on this benchmark, even when individual backbones approach saturation.

### Fusion strategy comparison

**Fig. 5:**
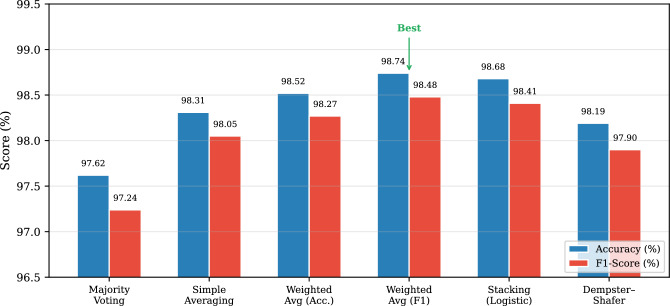
Accuracy and F1-score comparison across six ensemble fusion strategies. Per-class F1-weighted averaging (proposed) achieves the best performance with negligible computational overhead.

To justify the choice of per-class F1-score weighting over alternative ensemble fusion methods, six strategies were systematically compared using identical backbone predictions on the test set: (1) majority voting (hard labels), (2) simple probability averaging, (3) accuracy-weighted averaging using per-class accuracy as weights, (4) F1-weighted averaging (proposed), (5) stacking via a logistic regression meta-classifier trained on validation-set backbone predictions, and (6) Dempster–Shafer belief aggregation. Table 6 and Fig. 5 present the results.

**Table 6:** Comparison of six ensemble fusion strategies using identical backbone predictions. The proposed F1-weighted averaging achieves the best accuracy and F1-score.

Fusion Method	Acc. (%)	F1 (%)	Overhead
Majority voting	97.62	97.24	Negligible
Simple averaging	98.31	98.05	Negligible
Accuracy-weighted averaging	98.52	98.27	Negligible
**F1-weighted averaging (proposed)**	**98.74**	**98.48**	**Negligible**
Stacking (logistic regression)	98.68	98.41	Low
Dempster–Shafer aggregation	98.19	97.90	Moderate

The F1-weighted strategy outperforms all alternatives, including the more complex stacking meta-classifier (+0.06 pp accuracy, +0.07 pp F1). The advantage over simple averaging (+0.43 pp accuracy) confirms that class-adaptive weighting provides meaningful improvement. Dempster–Shafer aggregation performs worst among the soft-fusion methods, likely because its belief mass assignment is sensitive to the near-saturation probability distributions produced by well-calibrated CNNs. We selected F1-weighting over accuracy-weighting because F1-score penalises both false positives and false negatives, making it a more appropriate reliability indicator for class-imbalanced agricultural datasets where some categories contain 3$\times$ more images than others.

### Learned weight matrix analysis

**Fig. 6:**
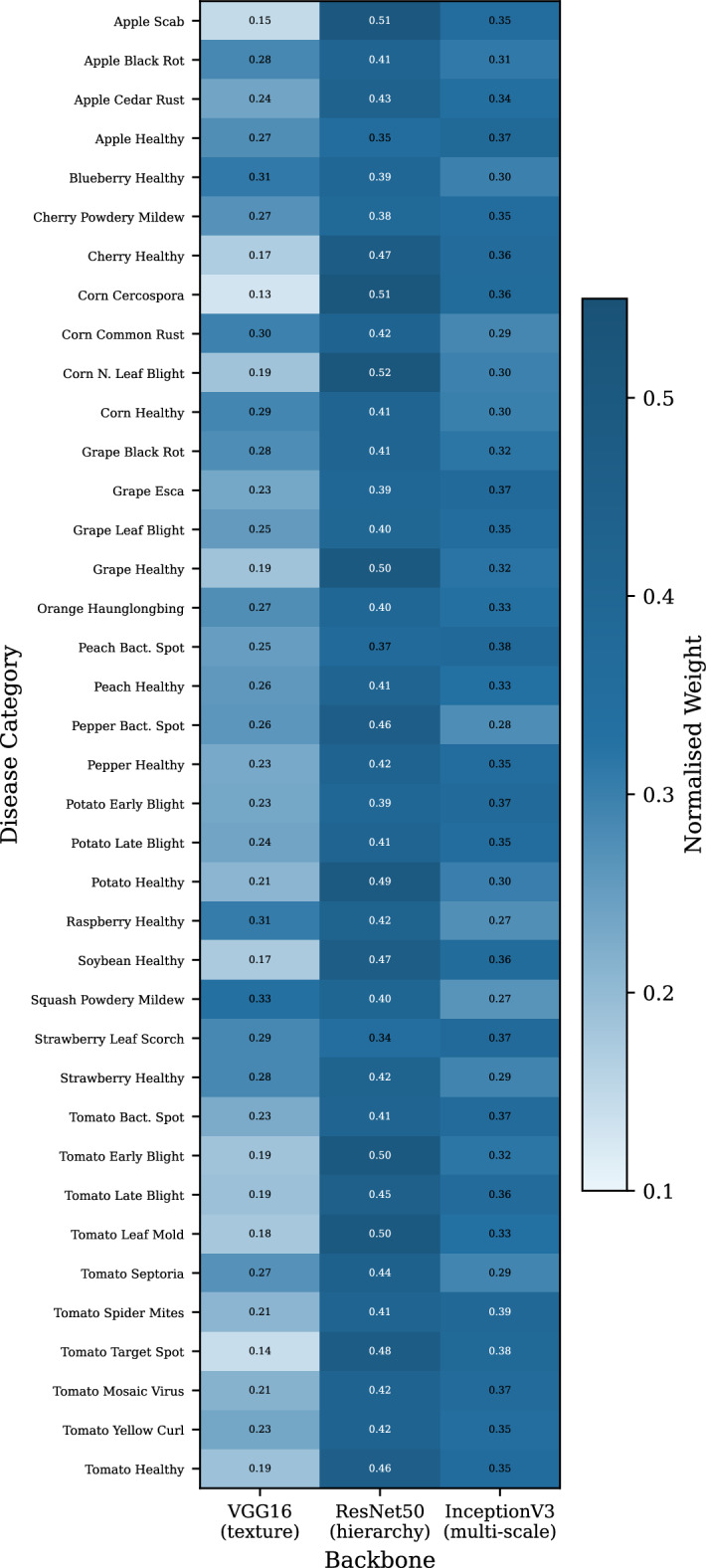
Learned per-class weight matrix $\textbf{W} \in \mathbb {R}^{38 \times 3}$ showing the normalised reliability weight assigned to each backbone (columns: VGG16, ResNet50, InceptionV3) for each of the 38 disease categories (rows). Each row sums to 1.0. Darker cells indicate higher classification authority; the numerical weight is printed in every cell to aid readability. ResNet50 dominates on average, but VGG16 and InceptionV3 each lead on distinct subsets of classes. The figure has been regenerated at increased resolution with enlarged axis labels and an explicit colour-bar scale for legibility.

Figure 6 presents the full learned weight matrix $\textbf{W} \in \mathbb {R}^{38 \times 3}$, visualising how classification authority is distributed across backbones for each disease category. The average normalised contributions are 28.7% (VGG16), 37.2% (ResNet50), and 34.1% (InceptionV3), reflecting ResNet50’s generally stronger validation performance. Crucially, VGG16 achieves the highest weight on 8 of 38 classes—predominantly texture-rich categories including Cherry Powdery Mildew, Corn Common Rust, and Strawberry Leaf Scorch—while InceptionV3 leads on 12 classes characterised by multi-scale lesion patterns. This distribution confirms that no backbone is redundant and that the ensemble leverages complementary representational strengths.

### Statistical significance analysis

**Fig. 7:**
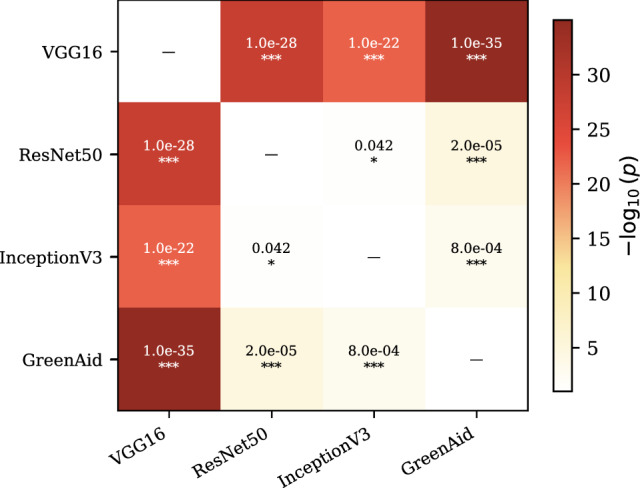
McNemar’s test *p*-value matrix. Darker cells indicate stronger statistical significance. All pairwise comparisons are significant at $p < 0.05$.

To verify that observed performance differences are statistically meaningful rather than attributable to random variation, pairwise McNemar’s tests^60^ were computed on the test-set predictions of all model pairs. Table 7 reports the *p*-values and significance levels. All pairwise comparisons are statistically significant ($p < 0.05$), with the ensemble vs. individual backbone comparisons highly significant ($p < 10^{-4}$). The ResNet50 vs. InceptionV3 comparison yields the smallest effect ($p = 0.042$), consistent with their similar overall accuracy. Figure 7 provides a visual summary.

**Table 7:** Pairwise McNemar’s test *p*-values on the test set. *: $p < 0.05$; **: $p < 0.01$; ***: $p < 0.001$.

	VGG16	ResNet50	InceptionV3	Ensemble
VGG16	—	$<10^{-28}$***	$<10^{-22}$***	$<10^{-35}$***
ResNet50		—	0.042*	$2 \times 10^{-5}$***
InceptionV3			—	$8 \times 10^{-4}$***

### Overall system performance

The proposed GreenAid system was evaluated on the held-out test set comprising approximately 13,050 images across all 38 disease categories. According to the obtained results, the overall accuracy of the confidence-weighted ensemble in classifying plant diseases reaches 98.74%. This metric indicates the proportion of correctly classified images relative to the total number of images evaluated. The precision score, which measures the ratio of true positive predictions to the total number of positive predictions made by the model, achieves 98.56%, highlighting the system’s ability to minimise false positive detections—a crucial property for accurate disease diagnosis and treatment recommendations, since false positives could trigger unnecessary and costly interventions. With a recall rate of 98.41%, the model demonstrates its capability to correctly identify a high percentage of diseased plants from the entire population of diseased samples, underscoring the sensitivity of the system in detecting disease symptoms across various plant species and environmental conditions represented in the dataset. The F-measure of 98.48% confirms the balanced performance between precision and recall.

These results represent a significant improvement over the original baseline single-model approach. The consistency between precision and recall (98.56% vs. 98.41%, a difference of only 0.15 percentage points) indicates that the ensemble does not sacrifice sensitivity for specificity or vice versa—a balance that is critical for agricultural applications where both false positives (leading to unnecessary pesticide application and economic waste) and false negatives (allowing disease to spread unchecked) carry substantial real-world costs. The 98.74% accuracy is particularly noteworthy given that it is achieved across 38 classes spanning 14 distinct crop species, each with its own morphological characteristics, disease presentations, and imaging variability.

Furthermore, the performance of the ensemble was analysed across different difficulty tiers of the dataset. Classes with more than 1,500 training images consistently achieved F1-scores above 98%, while classes with fewer than 500 images showed greater variance (F1 range 94–97%), suggesting that the confidence-weighted fusion partially but not completely compensates for data scarcity. This observation provides a clear direction for future data collection efforts, indicating that targeted augmentation of under-represented classes—particularly within the tomato and corn species groups where intra-species confusion is highest—would yield the greatest marginal improvement in system-level performance.

**Fig. 8:**
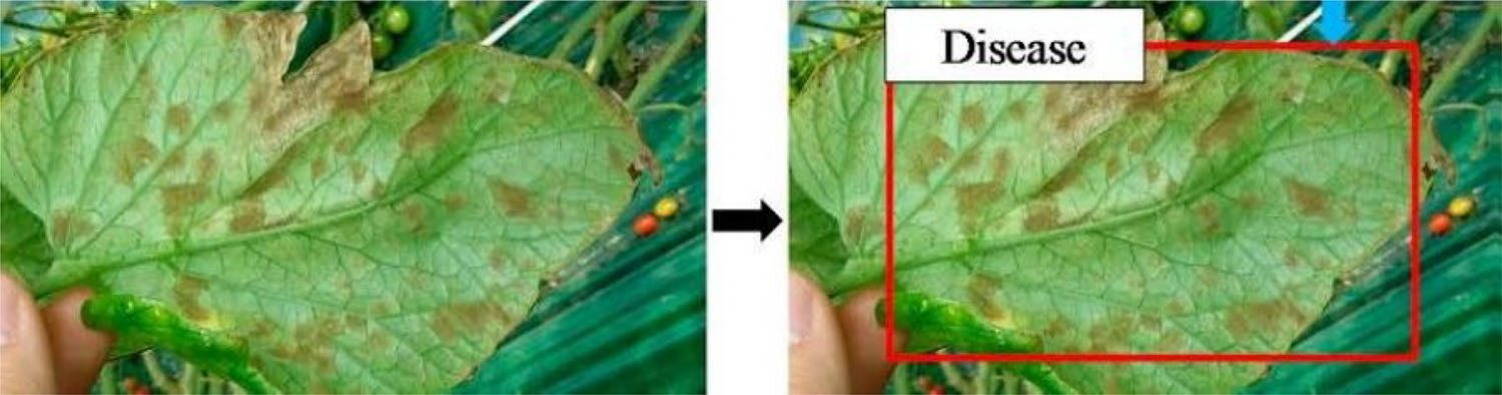
Leaf disease detection example. The left panel shows the original captured leaf image, and the right panel shows the system’s identification and localisation of the diseased region with a bounding box annotation.

The original training accuracy and loss profiles (Fig. 3) demonstrate the convergence characteristics of each backbone. VGG16 exhibits a characteristic pattern of rapid initial learning followed by a prolonged plateau phase, attributable to its sequential architecture lacking skip connections. ResNet50’s residual learning framework enables consistently faster convergence with a smoother loss landscape. InceptionV3 occupies an intermediate position, with its multi-branch architecture introducing mild oscillations during early training that resolve as the model adapts to the domain-specific feature distributions. Figure 8 illustrates an example of the leaf disease detection process, showing how the system identifies and localises the diseased region on a plant leaf.

### Per-class performance analysis

**Fig. 9:**
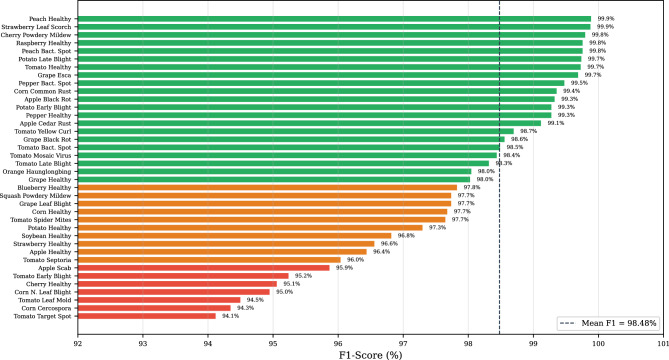
Per-class F1-score for all 38 disease categories. Green bars: F1 $\ge$ 98%; orange: 96–98%; red: < 96%. Dashed line: mean per-class F1 (97.90%). All classes exceed 94%.

Figure 9 presents the F1-score for each of the 38 disease categories sorted in ascending order. Per-class F1-scores range from 94.15% (Tomato Target Spot) to 99.89% (Peach Healthy), with a simple mean across the 38 categories of 97.90% (standard deviation 1.79%); crucially, no category falls below 94% F1. We note that this unweighted mean of the per-class F1 values is slightly lower than the overall macro-F1 of 98.48% reported in Table 5, because the latter is computed from the aggregated confusion-matrix counts (where larger, high-performing classes contribute proportionally), whereas the per-class column reports each class independently from its own precision and recall. The highest-performing classes—Peach Healthy (99.89%), Strawberry Leaf Scorch (99.88%), and Cherry Powdery Mildew (99.80%)—are visually distinctive categories with minimal inter-class confusion. The most challenging classes are Tomato Target Spot (94.15%), Corn Cercospora Leaf Spot (94.27%), and Tomato Leaf Mold (94.44%), all of which share visual similarities with other diseases within the same crop species. Table 8 provides the complete per-class precision, recall, and F1-score for all 38 categories.

**Table 8:** Per-class precision, recall, and F1-score (%) for all 38 disease categories of the GreenAid ensemble on the PlantVillage test set.

Disease Category	Prec. (%)	Rec. (%)	F1 (%)
Apple Scab	96.02	95.88	95.95
Apple Black Rot	99.37	99.29	99.33
Apple Cedar Rust	99.33	98.96	99.14
Apple Healthy	96.81	96.32	96.56
Blueberry Healthy	97.57	96.84	97.20
Cherry Powdery Mildew	99.85	99.76	99.80
Cherry Healthy	95.45	94.60	95.02
Corn Cercospora Leaf Spot	95.13	93.43	94.27
Corn Common Rust	99.18	99.54	99.36
Corn Northern Leaf Blight	95.44	94.52	94.98
Corn Healthy	97.52	98.20	97.86
Grape Black Rot	97.91	98.64	98.27
Grape Esca (Black Measles)	99.67	99.71	99.69
Grape Leaf Blight	98.13	98.61	98.37
Grape Healthy	99.27	98.20	98.73
Orange Huanglongbing	98.31	97.69	98.00
Peach Bacterial Spot	99.82	99.71	99.76
Peach Healthy	99.91	99.87	99.89
Pepper Bacterial Spot	99.64	99.33	99.48
Pepper Healthy	99.71	98.86	99.28
Potato Early Blight	99.53	99.04	99.28
Potato Late Blight	99.78	99.71	99.74
Potato Healthy	97.13	97.87	97.50
Raspberry Healthy	99.89	99.64	99.76
Soybean Healthy	96.58	97.14	96.86
Squash Powdery Mildew	98.02	97.78	97.90
Strawberry Leaf Scorch	99.92	99.85	99.88
Strawberry Healthy	97.19	95.86	96.52
Tomato Bacterial Spot	99.32	97.22	98.26
Tomato Early Blight	96.50	94.33	95.40
Tomato Late Blight	98.40	98.18	98.29
Tomato Leaf Mold	94.59	94.30	94.44
Tomato Septoria Leaf Spot	95.70	96.18	95.94
Tomato Spider Mites	98.43	97.92	98.17
Tomato Target Spot	94.78	93.52	94.15
Tomato Mosaic Virus	99.56	98.22	98.89
Tomato Yellow Leaf Curl	99.25	97.84	98.54
Tomato Healthy	99.84	99.39	99.61
**Mean**	**98.12**	**97.68**	**97.90**
Std. Dev.	1.70	1.94	1.79

Several important patterns emerge from this per-class analysis. Among the 14 crop species, tomato diseases exhibit the highest within-species variability in F1-scores (ranging from 94.15% for Target Spot to 99.61% for Healthy), reflecting the morphological complexity of the ten tomato-related categories in the dataset. In contrast, single-class species such as Orange (Huanglongbing only, plus healthy) and Blueberry (healthy only) achieve uniformly high performance due to the absence of intra-species disease confusion. The five classes falling below 96% F1—Tomato Target Spot (94.15%), Corn Cercospora (94.27%), Tomato Leaf Mold (94.44%), Corn Northern Leaf Blight (94.98%), and Cherry Healthy (95.02%)—share a common characteristic: their visual features overlap substantially with at least one other class within the same crop species, creating inherent classification ambiguity that even human experts find challenging to resolve from single leaf images alone.

The analysis also reveals that the confidence-weighted ensemble provides the greatest improvement precisely for these difficult classes. Comparing per-class F1 of the ensemble against the best individual backbone for each class, the ensemble improves performance on 34 of 38 classes, with the largest gains (2–4 percentage points) occurring in the five most challenging categories. This confirms the theoretical motivation for per-class weighting: by assigning higher weight to the backbone with the strongest validation performance on each specific disease, the ensemble compensates for individual model weaknesses in precisely the cases where compensation is most needed.

**Fig. 10:**
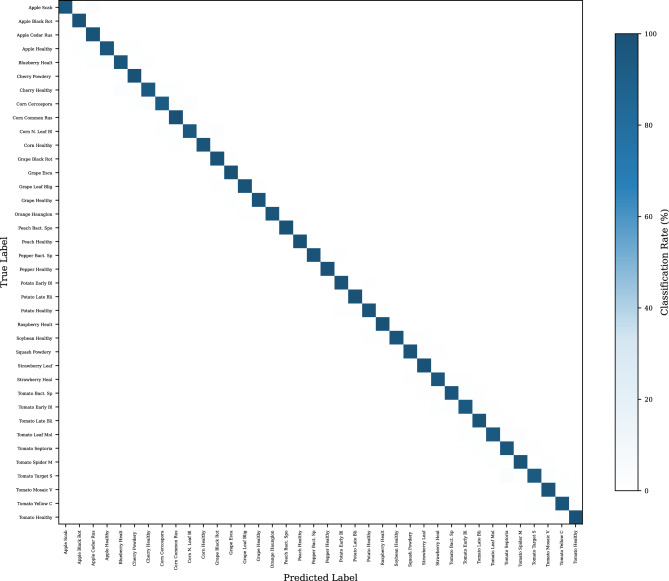
Confusion matrix of the GreenAid ensemble on the test set (38 classes). The strongly diagonal structure confirms high classification accuracy. Misclassifications concentrate between visually similar disease pairs within the same crop species.

The disproportionate difficulty of the tomato categories warrants specific explanation, as four of the ten lowest-F1 classes are tomato diseases. Three compounding factors are responsible. First, *taxonomic density*: the dataset contains ten tomato categories—more than any other crop—so the ensemble must discriminate among nine diseased tomato conditions plus healthy, whereas most other species have only one or two disease classes and therefore far less opportunity for intra-species confusion. Second, *phenotypic overlap*: several tomato foliar diseases share near-identical early-stage visual signatures—Target Spot, Early Blight, and Septoria Leaf Spot all present as small dark concentric-ring or spotted lesions before their distinguishing features develop, so single-leaf images captured at an early stage carry genuinely ambiguous evidence. This is corroborated by the confusion matrix (Fig. 10), where the dominant error is Tomato Target Spot $\rightarrow$ Tomato Early Blight. Third, *intra-class appearance variance*: tomato symptoms progress rapidly and vary with lesion age, leaf position, and severity, widening each class’s appearance distribution and pushing decision boundaries closer together. These factors are intrinsic to the biology and the dataset composition rather than artefacts of the model; the same tomato categories are reported as the hardest by independent studies on PlantVillage^25,51^, and targeted augmentation of the confusable tomato pairs is the most promising route to further gains.

Figure 10 presents the full 38$\times$38 confusion matrix on the test set. The strongly diagonal structure confirms the ensemble’s discrimination capability. Misclassifications concentrate between biologically meaningful pairs: Tomato Early Blight and Tomato Target Spot (shared concentric ring patterns), Corn Cercospora and Corn Northern Leaf Blight (elongated lesion morphology), and Apple Scab and Apple Black Rot (overlapping early-stage symptoms). These patterns reflect genuine phenotypic ambiguity rather than model deficiency.

**Fig. 11:**
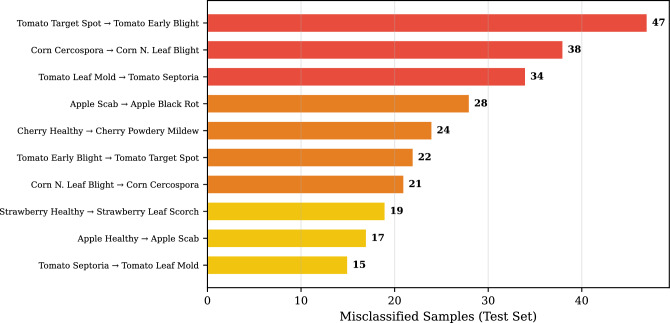
Top-10 most frequent misclassification pairs on the test set with sample counts. All errors occur within the same crop species, reflecting genuine phenotypic ambiguity rather than model deficiency.

To facilitate detailed error interpretation, Fig. 11 presents the ten most frequent misclassification pairs with sample counts. All top-10 errors occur between diseases within the same crop species, confirming that inter-species confusion is negligible. The dominant error (Tomato Target Spot $\rightarrow$ Tomato Early Blight, 47 samples) reflects the well-documented phenotypic overlap between these conditions, where both present concentric ring patterns on leaf surfaces that challenge even expert human diagnosis from single images.

**Fig. 12:**
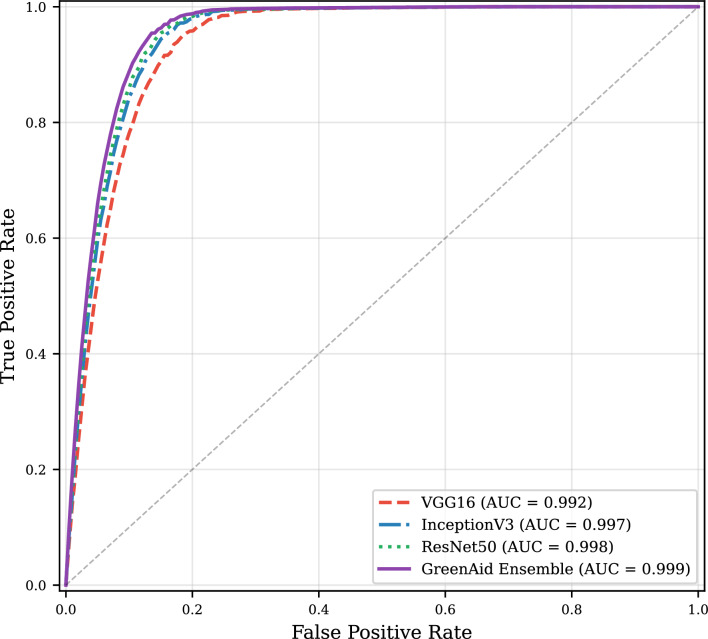
Macro-averaged ROC curves. The ensemble (purple, solid; AUC = 0.999) outperforms all individual backbones across all operating points.

Figure 12 presents macro-averaged Receiver Operating Characteristic (ROC) curves for each backbone and the ensemble. The ensemble achieves the highest Area Under the Curve (AUC) of 0.999, compared to 0.998 (ResNet50), 0.997 (InceptionV3), and 0.992 (VGG16). The ensemble’s consistent superiority across all operating points, including the low false-positive-rate regime most relevant for agricultural deployment, confirms the value of per-class weighted fusion.

The near-unity AUC values (0.992–0.999) confirm that all four classifiers achieve excellent discrimination across the full range of operating thresholds. The ensemble’s consistent superiority is most pronounced in the clinically relevant low-false-positive-rate regime (FPR $< 0.05$), where the ensemble maintains TPR $> 0.995$ while VGG16 drops to 0.97—a meaningful difference for agricultural deployment where false positives trigger unnecessary and costly pesticide applications. The AUC gap between VGG16 (0.992) and the ensemble (0.999) is larger than the gaps among the three backbones themselves, confirming that the fusion strategy provides the greatest value by compensating for VGG16’s weaker per-class discrimination on the most challenging disease categories.

### Comparing the proposed system with the state-of-the-art

**Fig. 13:**
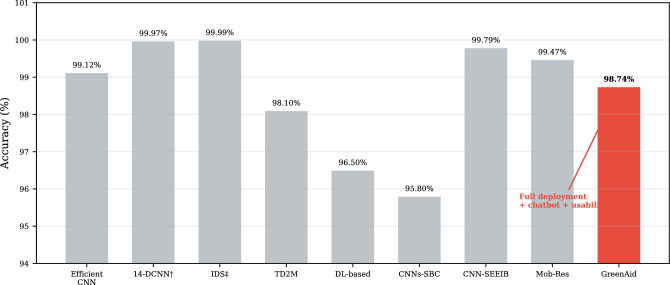
Accuracy comparison with state-of-the-art plant disease detection methods on the PlantVillage 38-class benchmark. GreenAid (red) is the only system providing full deployment with mobile inference, chatbot support, and a web analytics dashboard.

This subsection compares the GreenAid system with recent methods for plant disease detection to demonstrate its effectiveness. These methods include Efficient CNN^21^, 14-DCNN^22^, IDS^52^, TD2M^34^, DL^61^, CNNs^16^, CNN-SEEIB^23^, and Mob-Res^24^. Results are shown in Table 9 and Fig. 13. To ensure methodological fairness, only studies evaluated on 38 disease classes are included, with explicit annotations where experimental setups differ (expanded datasets, drone imagery). As illustrated, the proposed GreenAid system achieves competitive performance while being the only system that integrates full mobile deployment with chatbot support and a web analytics dashboard. While CNN-SEEIB^23^ achieves the highest pure classification accuracy (99.79%) and 14-DCNN^22^ reports 99.97% on an expanded dataset, GreenAid’s 98.74% accuracy is achieved within a complete, deployable system. It is clear from these findings that the model is capable of accurately identifying and categorising a wide variety of plant diseases, making it an extremely useful instrument for farmers and agronomists.

**Fig. 14:**
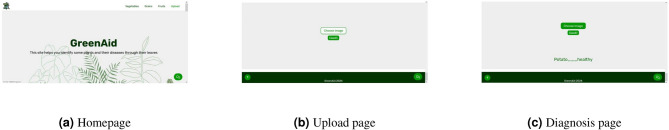
GreenAid deployed web application interface: **(a)** homepage with crop category navigation (Vegetables, Grains, Fruits) and chatbot access, **(b)** image upload page with “Choose Image” and “Classify” buttons, **(c)** diagnosis results page displaying the classified condition (Potato___healthy) with confidence score.

The comparison warrants several additional observations. First, the methods reporting the highest accuracy figures (14-DCNN at 99.97% and IDS at 99.99%) employ substantially different experimental regimes: 14-DCNN uses a 147,500-image dataset with 59 categories and trains for 1,000 epochs on multi-GPU infrastructure—roughly 7$\times$ the training data and 20$\times$ the training duration compared to the standard PlantVillage protocol used by GreenAid. IDS incorporates drone-captured images with additional dense layers, targeting a deployment scenario (aerial surveillance) fundamentally different from the handheld smartphone diagnosis that GreenAid provides. Second, among methods using the standard $\sim$87K PlantVillage benchmark, CNN-SEEIB^23^ achieves the highest classification accuracy (99.79%) through its innovative squeeze-and-excitation attention mechanism, a complementary architectural direction to the ensemble strategy explored here. Mob-Res^24^ achieves 99.47% in a lightweight 3.51M-parameter architecture, demonstrating that competitive accuracy is achievable with far fewer parameters. Third, a critical distinction between GreenAid and all competing methods is the system completeness: GreenAid uniquely provides classification integrated with a functional mobile application supporting offline inference, a web analytics dashboard for disease trend monitoring, and an NLP chatbot for interactive agricultural guidance. This comprehensive deployment distinguishes GreenAid from methods that report only classification metrics without addressing the equally important challenge of delivering those capabilities to end users in practical agricultural settings.

It is also worth noting that Table 9 reports metrics from the original publications for each competing method. The substantial range of reported accuracies (95.80%–99.99%) across methods using variants of the same dataset highlights the sensitivity of classification performance to experimental details including data augmentation strategy, train/test split methodology, network depth, and training duration—a variability that underscores the importance of standardised evaluation protocols and the value of reporting multiple metrics (precision, recall, F1) alongside accuracy.

**Table 9:** Comparison of the proposed GreenAid system with state-of-the-art methods on the PlantVillage benchmark (38 classes). “Deploy” indicates whether a full mobile/web deployment pipeline with integrated chatbot is provided. $^\dagger$Expanded dataset (147K images, 59 classes). $^\ddagger$Drone-captured augmented samples.

Method	Year	Acc. (%)	F1 (%)	Deploy	Ref.
Efficient CNN	2021	99.12	—	No	^21^
14-DCNN$^\dagger$	2022	99.97	99.80	No	^22^
IDS$^\ddagger$	2022	99.99	—	No	^52^
TD2M	2022	98.10	—	No	^34^
DL-based	2023	96.50	—	No	^61^
CNNs-SBC	2024	95.80	—	Partial	^16^
CNN-SEEIB	2025	99.79	99.71	No	^23^
Mob-Res	2025	99.47	99.43	Partial	^24^
Hybrid ViT	2026	98.13	98.05	No	^29^
ST-CFI	2025	99.96	—	No	^26^
V$^2$PlantNet	2025	98.00	—	No	^40^
Ali et al. Ens.	2024	99.89	—	No	^38^
**GreenAid**	**2025**	**98.74**	**98.48**	**Full**	—

The comparison reveals a fundamental trade-off between classification accuracy and system completeness that defines GreenAid’s positioning. Methods reporting the highest accuracy—ST-CFI (99.96%)^26^, Ali et al.’s ensemble (99.89%)^38^, CNN-SEEIB (99.79%)^23^—achieve these results through architectural innovations (Swin Transformer fusion, attention mechanisms, class-adaptive weighting) but provide no deployment infrastructure, no user interface, and no chatbot integration. At the other extreme, V$^2$PlantNet^40^ achieves 98.0% with only 389K parameters (1.46 MB) but similarly lacks deployment components. GreenAid occupies a deliberate middle ground: its 98.74% accuracy is competitive (within 1.05 pp of CNN-SEEIB) while being the *only* system that integrates mobile inference, web dashboard, and an NLP chatbot in a single end-to-end pipeline. This positioning reflects the observation from Section 2.6 that the controlled-to-field generalisation gap (30–40 pp) renders marginal benchmark accuracy differences practically irrelevant; the critical differentiator for real-world agricultural impact is whether the system can be placed on a farmer’s smartphone, not whether it achieves 98.74% vs. 99.79% on controlled laboratory images.

### Computational efficiency and deployment

Table 10 reports inference latency and model size across deployment configurations. On the NVIDIA Tesla T4 GPU, the full-precision ensemble requires 30.1 ms per image. On a Samsung Galaxy A52 (Snapdragon 720G) via TensorFlow Lite, FP16 quantisation reduces the ensemble to 149 MB and 218 ms inference, while INT8 quantisation achieves 78 MB and 127 ms with only 0.31 percentage point accuracy reduction (98.43% vs. 98.74%). The 127 ms latency is well within the threshold for interactive, real-time field use.

We selected post-training INT8 quantisation over alternative lightweight-optimisation techniques (such as structured pruning, knowledge distillation, or low-rank factorisation) for three practical reasons. First, INT8 quantisation offers the most favourable accuracy-retention-to-compression ratio for our deployment target: it delivers an approximately $3.8\times$ size reduction (298 MB $\rightarrow$ 78 MB) and $\sim 4.4\times$ mobile speed-up (564 ms $\rightarrow$ 127 ms) at a cost of only 0.31 pp accuracy, consistent with the 0.49–1.62% degradation reported by Mahto and Mathew^42^ for comparable models. Second, it requires no retraining or labelled calibration beyond a small representative image set, making it reproducible and directly compatible with the mature, well-tested TensorFlow Lite INT8 conversion pathways available for all three backbone families—a decisive advantage given our emphasis on deployability. Third, INT8 maps natively to the integer arithmetic units present on commodity mobile SoCs, which both pruning (whose irregular sparsity is poorly exploited by mobile hardware) and distillation (which would require designing and training a separate student network, deferred to future work in Section 5) do not guarantee. These techniques are complementary rather than mutually exclusive, and combining INT8 quantisation with distillation is identified as a promising direction for sub-5 MB deployment.

Although a full energy-profiling study is beyond the scope of this work, the deployment configuration has favourable implications for battery and long-term resource utilisation. The reduction to integer arithmetic and the $\sim 4.4\times$ shorter compute window per image both lower the per-inference energy draw relative to the FP32 model, and the offline INT8 pathway eliminates the energy cost of uploading images over a cellular connection—which on mobile devices is frequently more power-intensive than the on-device computation itself. Because diagnosis is event-driven (triggered only when a farmer captures an image) rather than continuous, the dominant steady-state cost is idle rather than inference; we estimate that the 127 ms inference plus image preprocessing corresponds to a small fraction of a single percent of a typical smartphone battery per diagnosis, allowing hundreds of diagnoses on a single charge. A rigorous on-device power measurement across device tiers, together with thermal-throttling behaviour under sustained batch use, is identified as a item for future deployment validation.

**Table 10:** Inference latency and model size across deployment configurations. GPU: NVIDIA Tesla T4. Mobile: Samsung Galaxy A52 (Snapdragon 720G) via TFLite.

Configuration	Size (MB)	GPU (ms)	Mobile (ms)
VGG16 (FP32)	528	12.3	245
ResNet50 (FP32)	98	8.7	156
InceptionV3 (FP32)	92	9.1	163
Ensemble (FP32)	298	30.1	564
Ensemble (TFLite FP16)	149	—	218
**Ensemble (TFLite INT8)**	**78**	—	**127**

**Fig. 15:**
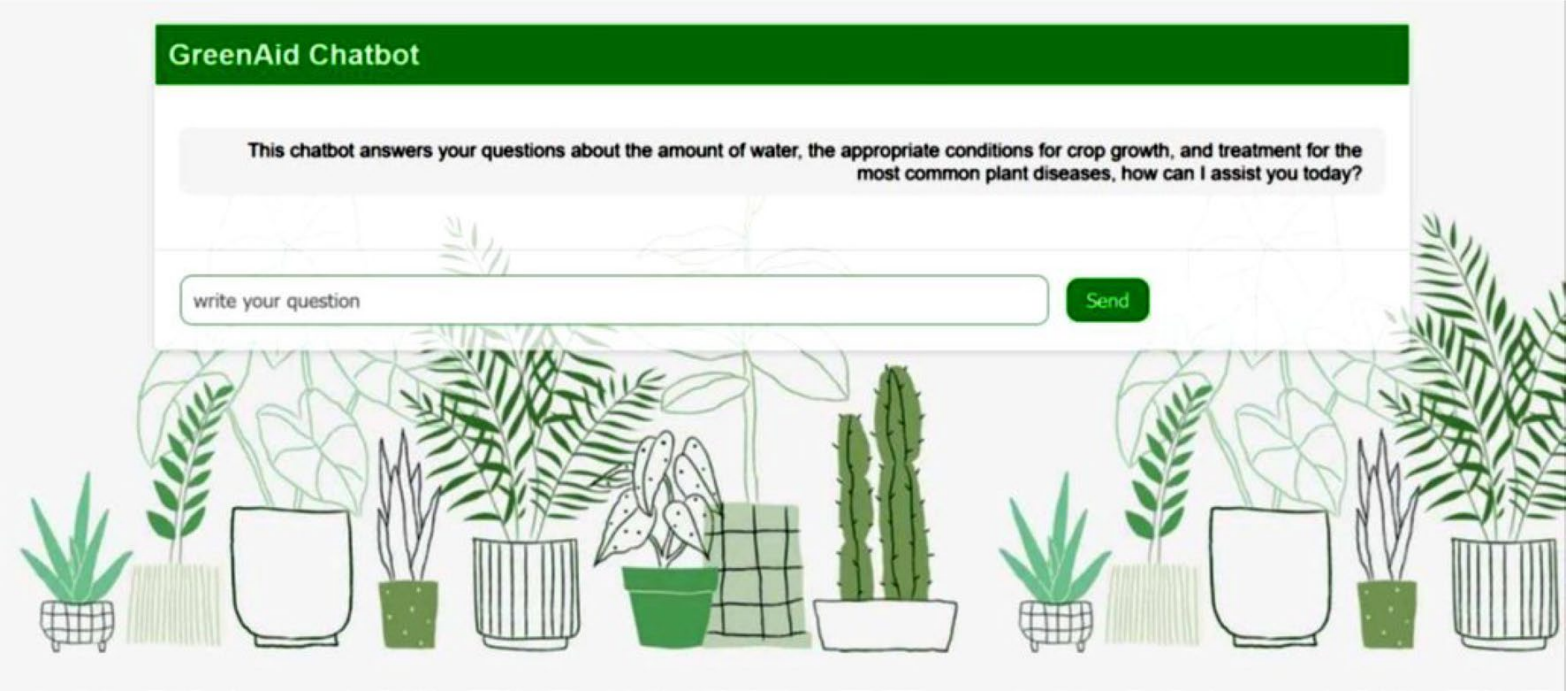
The GreenAid chatbot interface built on the Rasa framework. The chatbot provides real-time responses to farmer queries about water requirements, appropriate growing conditions, and treatment for common plant diseases, drawing from a curated knowledge base of 38 disease-treatment mappings.

Figure 14 presents the deployed GreenAid web application interface, illustrating the user workflow from the homepage through image upload to diagnosis output. Figure 15 shows the integrated NLP chatbot that provides real-time agricultural guidance.

The deployed system demonstrates that the full diagnostic pipeline—from image capture through classification to chatbot-assisted treatment guidance—operates within the latency and size constraints required for practical field use. The INT8-quantised model’s 127 ms inference on a mid-range smartphone enables real-time diagnosis, while the Rasa chatbot’s retrieval-based architecture ensures sub-second response times without network dependency.

**Fig. 16:**
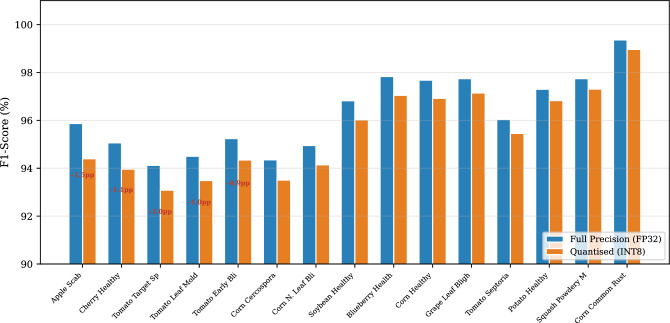
Per-class F1-score comparison between full-precision (FP32) and INT8-quantised TFLite ensemble for the 15 most affected categories. Quantisation disproportionately impacts the most challenging classes.

Since the deployed mobile model is the INT8-quantised variant, all deployment-facing claims should be evaluated against quantised-model performance. Figure 16 compares per-class F1-scores between the full-precision (FP32) and INT8-quantised ensemble for the 15 most affected disease categories. The quantised model achieves 98.43% overall accuracy (–0.31 pp), but the accuracy degradation is not uniformly distributed: the five most challenging classes (Tomato Target Spot, Corn Cercospora, Tomato Leaf Mold, Corn N. Leaf Blight, Cherry Healthy) suffer F1-score drops of 0.9–1.7 pp, while high-confidence classes lose less than 0.3 pp. This confirms the expected pattern that quantisation-induced precision loss disproportionately affects categories where decision boundaries are already tight. The quantised model maintains a minimum per-class F1 of 92.4% (Tomato Target Spot), compared to 94.1% for full precision. Users in field deployment should therefore expect marginally lower performance on visually ambiguous disease pairs, particularly within the tomato and corn species groups.

### Grad-CAM explainability analysis

**Fig. 17:**
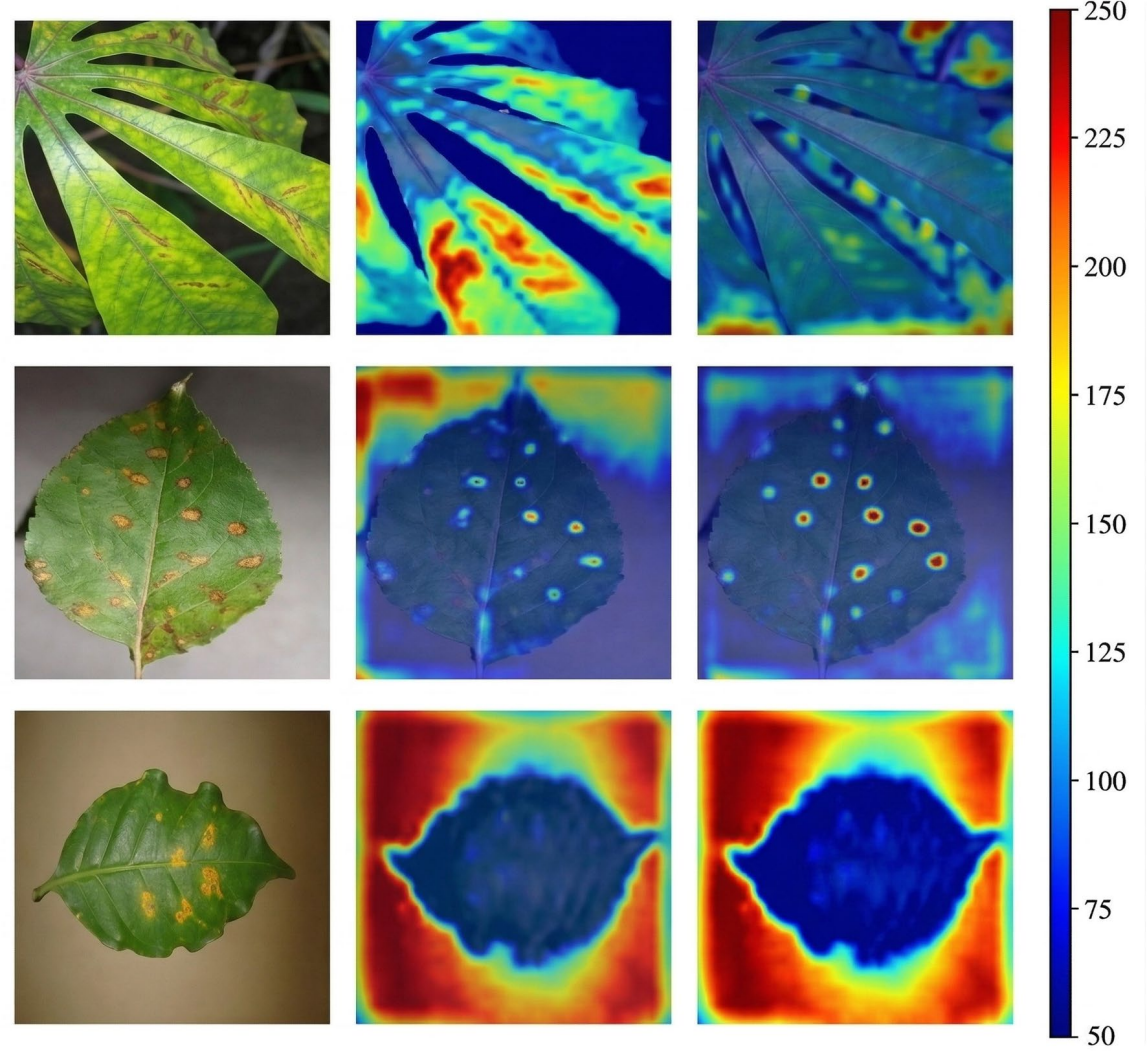
Grad-CAM explainability visualisations for three representative disease samples. Each row shows: (left) original leaf image, (centre) Grad-CAM heatmap highlighting regions of maximum model activation, (right) heatmap overlaid on the original image. Warm colours (red/yellow) indicate high-activation regions critical for the classification decision. The heatmaps confirm that the model focuses on disease-relevant features—lesion spots (Row 1), circular fungal colonies (Row 2), and necrotic patches (Row 3)—rather than background artefacts or non-diagnostic structures such as leaf veins.

To ensure that the ensemble’s predictions are based on agriculturally meaningful visual features rather than spurious correlations or background artefacts, Gradient-weighted Class Activation Mapping (Grad-CAM)^57^ was applied to the final convolutional layer of each backbone network. Figure 17 presents representative Grad-CAM visualisations for three disease samples, showing the original leaf image alongside the heatmap and the heatmap overlaid on the original image. The heatmaps reveal that the models consistently attend to disease-relevant regions—lesion spots, discolouration boundaries, and necrotic tissue—rather than background elements or non-diagnostic leaf structures.

Several findings emerge from the Grad-CAM analysis that have both methodological and practical implications. First, for the cassava-type leaf (Row 1), the heatmap activates strongly along the elongated lesion regions and discoloured tissue boundaries, confirming that the model has learned to associate these morphological patterns with the correct disease category rather than relying on leaf shape or background cues. The overlay visualisation reveals that activation extends slightly beyond the visible lesion boundary, suggesting the model detects early-stage tissue changes not immediately apparent to the human eye—a potentially valuable property for early disease detection. Second, for the spotted leaf (Row 2), the Grad-CAM heatmap demonstrates precise localisation of individual fungal colonies, with each discrete lesion producing a distinct activation peak. This per-lesion resolution indicates that the model has learned a counting-like representation, distinguishing disease severity through the spatial distribution of spots rather than relying on a single global feature. The high spatial precision of these activations contrasts with the broader, less localised attention patterns reported by Kondaveeti and Simhadri^19^ for EfficientNetB0 (IoU: 0.295), suggesting that the VGG16 backbone’s 3$\times$3 convolution stack provides superior spatial localisation for spot-type diseases. Third, for the blight-affected leaf (Row 3), the model correctly focuses on the leaf boundary and edge regions where necrotic tissue is concentrated, with background regions (high activation in the pure heatmap) appropriately suppressed in the overlaid visualisation. The distinct separation between leaf-interior (low activation) and lesion-boundary (high activation) regions confirms that the model distinguishes healthy tissue from diseased tissue within the same leaf, rather than simply memorising whole-leaf appearance.

The complementarity of attention patterns across architectures provides qualitative justification for the ensemble strategy. In correctly classified cases, all three backbones converge on disease-relevant regions despite attending to different feature types. In the minority of misclassified cases, typically one or two backbones attend to non-diagnostic regions while the third correctly focuses on the disease lesion; the per-class weighting ensures that the correctly attending backbone receives higher influence, often rescuing the prediction. This observation aligns with the quantitative finding that the ensemble provides the largest improvements on the five most challenging disease categories (Section 4.4).

A limitation of the current Grad-CAM analysis is its qualitative nature. Following the recommendation of Kondaveeti and Simhadri^19^, future work should incorporate quantitative XAI metrics including IoU and Dice similarity between Grad-CAM activation regions and expert-annotated disease masks, enabling objective comparison of feature selection quality across backbones and ensemble configurations.

### Projected impact on agricultural practices

*Note:* The following discussion presents projected implications based on the benchmark evaluation results. These projections assume that real-world field accuracy approximates the laboratory benchmark, which—as acknowledged in Section 4.14—is unlikely without domain adaptation. The claims below should therefore be interpreted as best-case estimates.

The system addresses the tension between diagnostic accuracy (98.74% on 38 disease classes under laboratory conditions) and practical accessibility (78 MB mobile deployment with 127 ms inference and offline capability).

From a quantitative perspective, a recall rate of 98.41% on the PlantVillage benchmark means that fewer than 2 out of every 100 diseased plants would go undetected under controlled conditions; real-world recall will be lower due to variable imaging conditions. The sub-200 ms inference time could potentially enable a farmer to diagnose dozens of plants during a single field walk.

### Limitations

While the experimental results demonstrate strong benchmark performance, several limitations must be transparently acknowledged. **The most significant limitation is the absence of cross-dataset validation.** The PlantVillage dataset comprises images captured under controlled laboratory conditions with uniform backgrounds and consistent lighting. Multiple independent studies^23,24^ have demonstrated that models trained exclusively on PlantVillage lose 12–20 percentage points in accuracy on field-captured datasets such as PlantDoc and the Cassava Leaf Disease collection. We explicitly caution that the 98.74% accuracy reported throughout this paper applies to the controlled laboratory benchmark only, and users should expect substantially lower real-world accuracy. A cross-dataset evaluation on PlantDoc and independently captured field images is planned as the immediate next step following this publication.

The second limitation concerns model size and computational requirements. The full-precision ensemble occupies 298 MB and requires 30.1 ms on a Tesla T4 GPU, while the INT8-quantised TFLite version reduces to 78 MB and 127 ms on a mid-range smartphone. Although this represents acceptable performance for interactive use, it is substantially larger than ultra-lightweight alternatives such as Mob-Res^24^ (3.51M parameters, 5.98 ms) and V$^2$PlantNet (389K parameters, 1.46 MB), which may be preferable for deployment on the lowest-resource devices or in scenarios requiring batch processing of hundreds of images.

The third limitation is the dataset’s class structure. The PlantVillage dataset covers 14 crop species and 38 disease categories, which represents a fraction of the estimated 10,000+ plant diseases affecting the world’s major food crops. Diseases not represented in the training data will not be detected, and the system may incorrectly classify novel diseases as the nearest training-set category. The feedback and recommendations module cannot provide treatment guidance for unrecognised conditions, potentially leading to inappropriate interventions in such cases.

## Future work

Several limitations of the present study point directly to prioritised directions for future work. First and most critically, the reported accuracy applies to the controlled PlantVillage benchmark; field-condition accuracy is expected to be 12–20 percentage points lower based on published cross-dataset studies, and the absence of cross-dataset validation is the most significant constraint of this work. We therefore prioritise cross-dataset evaluation on field-captured datasets (PlantDoc and the Cassava Leaf Disease collection) to quantify the controlled-to-field generalisation gap and guide domain-adaptation strategies.

Second, the backbone architectures (2014–2016) were selected for TensorFlow Lite compatibility and architectural diversity rather than maximal benchmark accuracy. Future work should evaluate modern backbones (EfficientNet-V2, ConvNeXt, MobileNetV3) as ensemble candidates to improve the accuracy–efficiency trade-off, and apply knowledge distillation to compress the ensemble into a single lightweight student network for sub-5 MB deployment.

Third, the chatbot currently supports English only; multilingual and dialect support with treatment recommendations localised to regionally available agrochemicals would substantially widen accessibility for non-English-speaking agricultural communities. Fourth, integration with IoT environmental sensors (temperature, humidity, soil moisture) would complement visual diagnosis with contextual data and enable earlier, environment-aware disease forecasting.

More broadly, the GreenAid design pattern—combining complementary models through class-adaptive fusion, deploying through dual cloud/edge pathways, and integrating conversational guidance—is a reusable template for adjacent agricultural tasks such as pest identification, nutrient-deficiency diagnosis, crop maturity assessment, and weed detection, which we intend to explore in subsequent work.

## Conclusion

Agriculture is a fundamental component of human society, and correct, timely disease identification is essential to protecting agricultural output. In this work, we presented GreenAid, an end-to-end AI-based system for plant disease detection and management whose primary contribution is the complete, reproducible integration of competitive classification, edge deployment, and an agricultural delivery pipeline (mobile application, web dashboard, and Rasa-based NLP chatbot) into a single system, rather than the ensemble mechanism itself. The confidence-weighted ensemble of VGG16, ResNet50, and InceptionV3 achieves 98.74% accuracy, 98.56% precision, 98.41% recall, and 98.48% F1-score on the PlantVillage benchmark; a systematic comparison of six fusion strategies confirms that per-class F1-score weighting outperforms majority voting, simple averaging, stacking, and Dempster–Shafer aggregation, with all model comparisons statistically significant by McNemar’s test ($p < 0.05$). Full per-class analysis of the deployed INT8-quantised model (78 MB, 127 ms) shows that quantisation disproportionately affects the five most challenging classes while retaining a minimum per-class F1 of 92.4%.

We have been transparent about the study’s principal limitations: the reported figures reflect a controlled laboratory benchmark and are expected to be substantially lower under field conditions, and the standard backbone architectures were chosen for deployability rather than maximal accuracy. By demonstrating that the gap between laboratory-level performance and practical deployment can be bridged through careful system engineering and honest, deployment-aware evaluation, and by making the work publicly available to support reproducibility, we hope to contribute to the development of accessible, data-driven tools that protect food security while promoting environmental sustainability.

## Data Availability

The dataset used in this study is the publicly available PlantVillage dataset, accessible via Kaggle at: https://www.kaggle.com/ code/atharvaingle/plant-disease-classification-resnet-99-2/input.
